# Human IL-23 is essential for IFN-γ-dependent immunity to mycobacteria

**DOI:** 10.1126/sciimmunol.abq5204

**Published:** 2023-02-10

**Authors:** Quentin Philippot, Masato Ogishi, Jonathan Bohlen, Julia Puchan, Andrés Augusto Arias, Tina Nguyen, Marta Martin-Fernandez, Clement Conil, Darawan Rinchai, Mana Momenilandi, Alireza Mahdaviani, Mohammad Keramatipour, Jérémie Rosain, Rui Yang, Taushif Khan, Anna-Lena Neehus, Marie Materna, Ji Eun Han, Jessica Peel, Federico Mele, Marc Weisshaar, Sandra Jovic, Paul Bastard, Romain Lévy, Tom Le Voyer, Peng Zhang, Majistor Raj Luxman Maglorius Renkilaraj, Carlos A. Arango-Franco, Simon Pelham, Yoann Seeleuthner, Mathieu Pochon, Manar Mahmoud Ahmad Ata, Fatima Al Ali, Mélanie Migaud, Camille Soudée, Tatiana Kochetkov, Anne Molitor, Raphael Carapito, Seiamak Bahram, Bertrand Boisson, Claire Fieschi, Davood Mansouri, Nico Marr, Satoshi Okada, Mohammad Shahrooei, Nima Parvaneh, Zahra Chavoshzadeh, Aurélie Cobat, Dusan Bogunovic, Laurent Abel, Stuart Tangye, Cindy S. Ma, Vivien Béziat, Federica Sallusto, Stéphanie Boisson-Dupuis, Jacinta Bustamante, Jean-Laurent Casanova, Anne Puel

**Affiliations:** 1 Laboratory of Human Genetics of Infectious Diseases, Necker Branch, INSERM U1163, Necker Hospital for Sick Children, Paris, France; 2 University Paris Cité, Imagine Institute, Paris, France; 3 St Giles Laboratory of Human Genetics of Infectious Diseases, Rockefeller Branch, Rockefeller University, New York, NY, USA; 4 Institute of Microbiology, ETH Zürich, Zurich, Switzerland; 5 Primary Immunodeficiencies Group, University of Antioquia UdeA, Medellin, Colombia; 6 School of Microbiology, University of Antioquia UdeA, Medellin, Colombia; 7 Garvan Institute of Medical Research, Darlinghurst, Australia; 8 St. Vincent’s Clinical School, Faculty of Medicine & Health, UNSW Sydney, Darlinghurst, Australia; 9 Center for Inborn Errors of Immunity, Icahn School of Medicine at Mount Sinai, New York, NY, USA; 10 Precision Immunology Institute, Icahn School of Medicine at Mount Sinai, New York, NY, USA; 11 Mindich Child Health and Development Institute, Icahn School of Medicine at Mount Sinai, New York, NY, USA; 12 Department of Pediatrics, Icahn School of Medicine at Mount Sinai, New York, NY, USA; 13 Department of Microbiology, Icahn School of Medicine at Mount Sinai, New York, NY, USA; 14 Pediatric Respiratory Diseases Research Center, National Research Institute of Tuberculosis and Lung Diseases (NRITLD), Shahid Beheshti University of Medical Sciences, Tehran, Iran.; 15 Department of Medical Genetics, School of Medicine, Tehran University of Medical Sciences, Tehran, Iran; 16 Department of Human Immunology, Sidra Medicine, Doha, Qatar; 17 Institute for Research in Biomedicine, Università della Svizzera italiana, Bellinzona, Switzerland.; 18 Department of Pediatrics, Necker Hospital for Sick Children, Paris, France; 19 Laboratoire d’ImmunoRhumatologie Moléculaire, Institut National de la Santé et de la Recherche Médicale (INSERM) UMR_S1109, Plateforme GENOMAX, Faculté de Médecine, Fédération Hospitalo-Universitaire OMICARE, Centre de Recherche d’Immunologie et d’Hématologie, Centre de Recherche en Biomédecine de Strasbourg (CRBS), Fédération de Médecine Translationnelle de Strasbourg (FMTS), Université de Strasbourg, Strasbourg, France; 20 Institut Thématique Interdisciplinaire (ITI) de Médecine de Précision de Strasbourg, Strasbourg, France; 21 Laboratoire d’Immunologie, Plateau Technique de Biologie, Pôle de Biologie, Nouvel Hôpital Civil, Strasbourg, France; 22 Clinical Immunology Department, Saint Louis Hospital, Paris, France; 23 Clinical Tuberculosis and Epidemiology Research Centre, National Research Institute of Tuberculosis and Lung Diseases (NRITLD), Shahid Beheshti University of Medical Sciences, Tehran, Iran; 24 College of Health and Life Sciences, Hamad Bin Khalifa University, Doha Qatar; 25 Department of Pediatrics, Hiroshima University Graduate School of Biomedical and Health Sciences, 1-2-3 Kasumi, Minami-Ku, Hiroshima-Shi, Hiroshima, Japan; 26 University of Leuven, Leuven, Belgium; 27 Research Center for Immunodeficiencies, Pediatrics Center of Excellence, Children’s Medical Center, Teheran University of Medical Sciences, Teheran, Iran; 28 Pediatric Infections Research Center, Mofid Children’s Hospital, Shahid Beheshti University of Medical Sciences, Tehran, Iran; 29 Study Center for Primary Immunodeficiencies, Necker Hospital for Sick Children, AP-HP, Paris, France; 30 Howard Hughes Medical Institute, New York, NY, USA

## Abstract

Patients with autosomal recessive (AR) IL-12p40 or IL-12Rβ1 deficiency display Mendelian susceptibility to mycobacterial disease (MSMD) due to impaired IFN-γ production and, less commonly, chronic mucocutaneous candidiasis (CMC) due to impaired IL-17A/F production. We report six patients from four kindreds with AR IL-23R deficiency. These patients are homozygous for one of four different loss-of-function *IL23R* variants. All six patients have a history of MSMD but only two suffered from CMC. We show that IL-23 induces IL-17A only in MAIT cells, possibly contributing to the incomplete penetrance of CMC in patients unresponsive to IL-23. By contrast, IL-23 is required for both baseline and *Mycobacterium*-inducible IFN-γ immunity in both Vδ2^+^ γδ T and MAIT cells, probably contributing to the higher penetrance of MSMD in these patients. Human IL-23 appears to contribute to IL-17A/F-dependent immunity to *Candida* in a single lymphocyte subset, but is required for IFN-γ-dependent immunity to *Mycobacterium* in at least two lymphocyte subsets.

## One-Sentence Summary

IL-23 signaling is required for baseline and IL-23-inducible IFN-γ immunity in both Vδ2^+^ γδ T and MAIT cells.

## INTRODUCTION

Life-threatening disease during primary infection in otherwise healthy individuals can result from monogenic inborn errors of immunity (IEI) (1, 2). Studies of such IEIs can shed light on the essential and redundant roles of the corresponding human genes in host defense *in natura,* while clarifying mechanisms of disease (3–6). Mendelian susceptibility to mycobacterial disease (MSMD) is characterized by selective susceptibility to clinical disease caused by weakly virulent mycobacteria, such as *Mycobacterium bovis*-Bacillus Calmette-Guérin (BCG) vaccine and environmental mycobacteria (7). Patients with MSMD are also prone to *bona fide* tuberculosis (TB), and to severe disease caused by *Salmonella* and, more rarely, other intra-macrophagic bacteria, fungi, and parasites (8). The genetic dissection of MSMD has led to the discovery of 37 genetic disorders, involving 19 genes, with allelic heterogeneity. All but one of these genetic disorders clearly affect interferon-γ (IFN-γ)-mediated immunity, the exception being ZNFX1 deficiency, the pathogenic mechanism of which remains unclear (7, 9–14).

The two most common etiologies of MSMD are autosomal recessive (AR) complete IL-12Rβ1 and IL-12p40 deficiencies. Since 1998, these deficiencies have been reported in more than 400 and 100 patients, respectively (15–24). AR complete IL-12p40 deficiency is a clinical phenocopy of IL-12Rβ1 deficiency (19, 24–27). IL-12p40 dimerizes with IL-12p35 to form IL-12, or with IL-23p19 to form IL-23 (28, 29). IL-12Rβ1 dimerizes with IL-12Rβ2 to form the IL-12 receptor, or with IL-23R to form the IL-23 receptor (30, 31). Consequently, both IL-12- and IL-23-mediated immunity are abolished in patients with IL-12Rβ1 or IL-12p40 deficiency (11). Patients with IL-12Rβ1 or IL-12p40 deficiency display incomplete penetrance for MSMD, with about a third of adult patients remaining MSMD-free; the penetrance for TB in endemic areas is probably higher, as *Mycobacterium tuberculosis* is at least 1,000 times more virulent than BCG and environmental mycobacteria (7, 8, 18, 19, 22, 32–34).

About 25% of patients with IL-12Rβ1 or IL-12p40 deficiency also suffer from chronic mucocutaneous candidiasis (CMC) (8, 18, 19, 35, 36). CMC is characterized by recurrent lesions of the skin, nails, and oral and genital mucosae caused by *Candida spp.* (36). This condition is not observed in patients with most other genetic etiologies of MSMD, including those in whom IFN-γ-mediated immunity is completely abolished (7, 37–40). The occurrence of isolated CMC in patients with autosomal dominant (AD) IL-17F deficiency or inborn errors of the IL-17-responsive pathway, such as AR IL-17RA, IL-17RC, and ACT1, and AD JNK1 deficiencies, revealed the essential role of IL-17A/F in mediating mucocutaneous immunity to *Candida albicans (C. albicans)* (41–45). IL-12Rβ1-deficient patients have low counts of circulating T helper 17 (T_H_17) cells, which has been attributed to the abolition of IL-23-dependent IL-17 cytokine induction in these patients (46). Likewise, patients with RORγT deficiency display impaired IFN-γ and IL-17A/F production and suffer from both MSMD and CMC (47).

In 2018, we reported AR IL-12Rb2 deficiency in three individuals from the same family: one with MSMD, another with TB, and an asymptomatic individual (11). More surprisingly, we reported AR IL-23R deficiency in two siblings with MSMD but not CMC (11). Together with the report that homozygosity for the common P1104A *TYK2* allele selectively impairs cellular responses to IL-23 and underlies TB and MSMD with high and low penetrance, respectively (9, 12, 48), these findings suggested that human IL-23 is required for optimal IFN-γ-dependent immunity to mycobacteria, but redundant for optimal IL-17-dependent immunity to *C. albicans* (11). This finding is intriguing in two ways, as the T_H_1/T_H_17 dichotomy had long revolved around the idea that IL-12 is a signature inducer of IFN-γ, whereas IL-23 is a signature inducer of IL-17A/F (49–52). We investigated the role of human IL-23 in host defense further, by searching for new patients with AR IL-23R deficiency in a cohort of >15,000 patients with diverse infectious diseases.

## RESULTS

### Six patients from four kindreds homozygous for private *IL23R* variants

**Figure 1:**
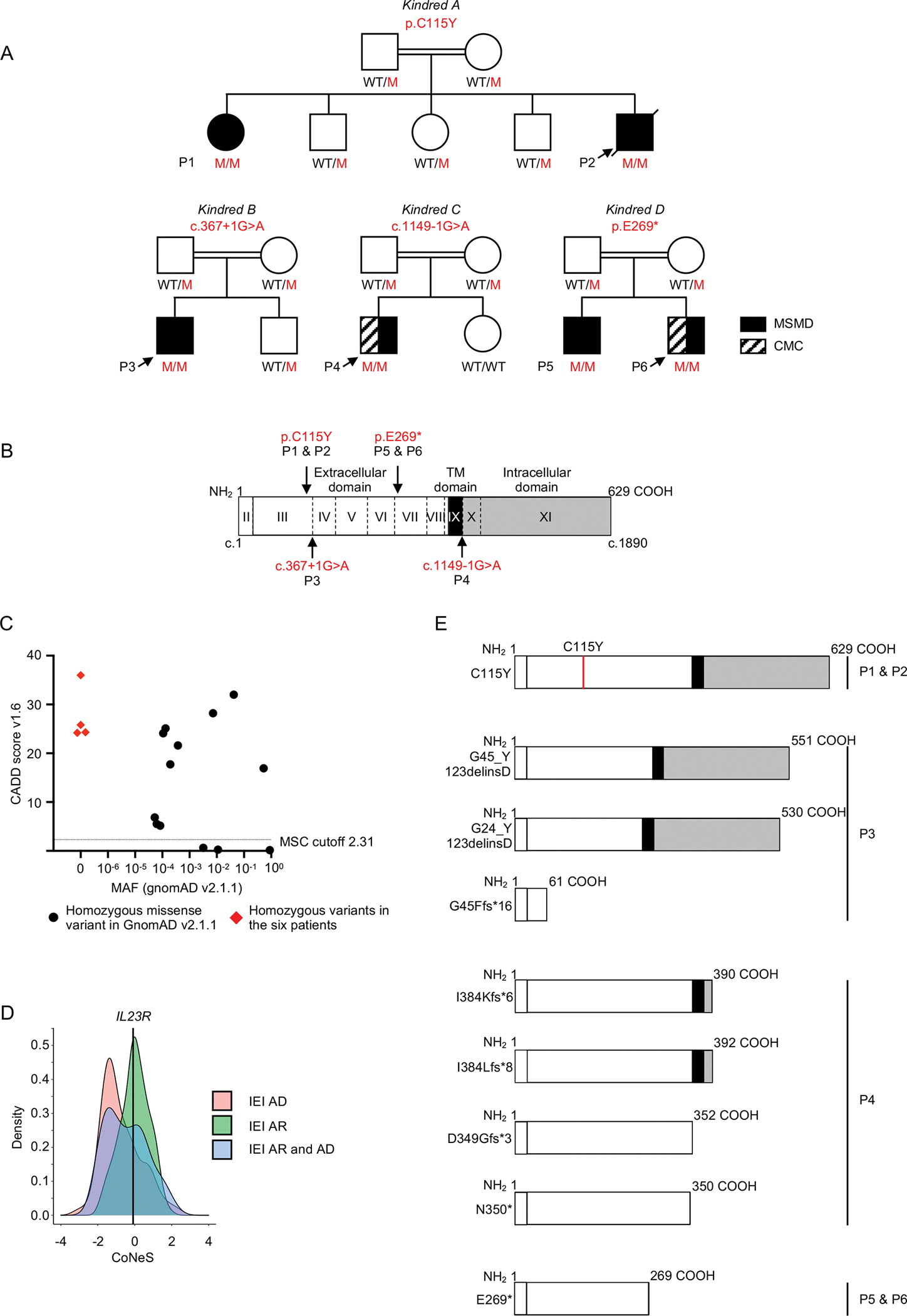
Six patients from four kindreds with private homozygous *IL23R* variants **(A)** Pedigrees of the four kindreds studied here. The *IL23* variants are indicated below the kindred name. Solid black symbols indicate patients with MSMD, and black diagonal stripes indicate CMC. Symbols linked with a double line indicate consanguinity. The genotype is indicated under each symbol, with M corresponding to the variant found in each kindred, and WT indicating wild-type. Arrows indicate the index case in each family. (**B**) Schematic representation of the WT IL-23R gene/protein, the colorless area represents the extracellular domain, the black area the transmembrane domain and the gray area the intracellular domain of the protein. The positions of the variants found in the patients are indicated by arrows. (**C**) All homozygous variants found in gnomAD V2.1.1 (in black) for *IL23R* are plotted according to their combined annotation-dependent depletion (CADD score; *y* axis) and minor allele frequency (MAF; *x* axis). The dashed line indicates the mutation significance cutoff (MSC) for *IL23R*. (**D**) Gene14 level negative selection. *IL23R* is not under negative selection, like other genes for which mutations underlie AR inborn errors of immunity (IEI), as determined by CoNeS. (**E**) Schematic representation of the impact of the different variants of the patients (P1-P6) on IL- 23R.

We searched for non-synonymous, essential splice site, or copy number variants of *IL23R* in a cohort of >15,000 patients with highly diverse infectious diseases, including 1,618 patients with MSMD, 1,454 with TB, and 730 with CMC. The cohorts overlap due to the occurrence of syndromic forms of infection. We selected rare variants (MAF<0.01) that were homozygous or potentially compound heterozygous. Six patients from four kindreds were homozygous for private *IL23R* variants (NM_144701.2) (Figure 1A). P1 and P2 from Kindred A, reported in previous studies (11, 22), are homozygous for the c.344G>A missense variant, which results in the p.C115Y substitution in a key domain of IL-23R (Figure 1B) (11, 53). P3, from Kindred B, is homozygous for the c.367+1G>A variant, which is predicted to alter the canonical donor splice site of exon 3 (Figure 1A and 1B). P4 from Kindred C, is homozygous for the c.1149–1G>A variant, which is predicted to alter the canonical acceptor splice site of exon 10 (Figure 1A and 1B). Finally, P5 and P6, from Kindred D, are homozygous for the nonsense c.805G>T variant (p.E269*) upstream from the segment encoding the transmembrane domain of IL-23R (Figure 1A and 1B) (53). All heterozygous or homozygous wild-type (WT) relatives of these patients were healthy (Figure 1A). The four *IL23R* variants found in kindreds A-D are not reported in any individuals of the Genome Aggregation Database (gnomAD) v2.1.1 (https://gnomad.broadinstitute.org/), ATAVDB (http://atavdb.org), Great Middle East Database (http://igm.ucsd.edu/gme), the Iranome database (http://www.iranome.com/), or in any other individuals from our in-house database (>15,000 individuals). Finally, these four *IL23R* variants are proven (p.C115Y) or predicted to be loss-of-function (pLOF) (c.367+1G>A, c.1149–1G>A, p.E269*), with combined annotation-dependent depletion (CADD) scores well above the mutation significance cutoff (MSC) of *IL23R* (Figure 1C) (54–56). These data suggest that these six patients have AR IL-23R deficiency.

### Six patients with MSMD, including two with CMC

The six patients belonged to four unrelated families originating from and living in Iran. Principal component analysis on whole-exome sequencing (WES) data confirmed the Iranian ancestry of P5 and P6, whereas P1 to P4 were closer to the European population (Figure S1A) (57). The high rate of homozygosity in these patients was suggestive of familial consanguinity (Figure S1B) (58), and their kinship coefficients suggested that the four kindreds were not related to each other (59). P1 (Kindred A), born in 1994 to consanguineous parents, was vaccinated with BCG in early infancy and developed BCG-adenitis (BCG-itis), which persisted for one year before spontaneously resolving (Table S1). Her brother, P2, born in 2006, was also vaccinated with BCG early in infancy, leading to BCG-itis, which progressed to disseminated BCG disease (BCG-osis) despite antibiotic treatment, resulting in the death of this patient at the age of eight years. P3 (Kindred B), born in 2010 to consanguineous parents, was vaccinated with BCG and developed BCG-osis (22). P4 (Kindred C), born in 2008 to consanguineous parents, was vaccinated with BCG and developed BCG-itis, which persisted for three years before spontaneously resolving. P4 has also suffered from oral and esophageal candidiasis, which persisted between the ages of three months and ten years. He was treated with fluconazole, and no recurrence has been observed on prophylaxis. P5 (Kindred D), born in 2015 to consanguineous parents, was vaccinated with BCG and developed axillary BCG adenitis (BCG-itis) at eight months, with spontaneous resolution at 12 months. His brother P6, born in 2019, was vaccinated with BCG and developed recurrent axillary BCG-itis, which resolved at 12 months after abscess drainage and antimycobacterial therapy until the age of 18 months. P6 has also presented one episode of oral candidiasis, which resolved on fluconazole. No infections with other intramacrophagic pathogens, such as environmental mycobacteria and *Salmonella*, were observed in any of the six patients, despite such infections being common in patients with IL-12Rβ1 and IL-12p40 deficiencies. Clinical penetrance was complete for BCG disease, but incomplete for CMC (two of the six patients) (Table S1). All six patients have been infected with diverse viruses and bacteria (Figure S1C), without severe clinical consequences. WES analysis of the six patients found no other homozygous candidate variants of known MSMD- or CMC-causing genes. Other rare homozygous or hemizygous coding and splice-site (including essential splice site) variants were present, with CADD scores above the corresponding MSC scores, and these variants were not present in the homozygous or hemizygous state in GnomAD v2.1.1. However, none of these variants were related to IFN-γ or IL-17 immunity (Figure S1B and Table S2). Overall, the six patients with a putative diagnosis of AR IL-23R deficiency had an MSMD phenotype, and two also had CMC.

### Population genetics of the *IL23R* locus

No *IL23R* pLOF variants were found in the homozygous state in the general population. Only 13 in-frame variants were found in the homozygous state; all were missense variants with MAFs ranging from 5.3 × 10^−5^ to 8.8 × 10^−1^ (Figure 1C). We reported all of these variants to be neutral in an overexpression assay in a previous study (11). A revised analysis of the population genetics of *IL23R* showed that the situation had not changed since 2018. The *IL23R* locus is subject to negative selection, with a consensus-based measure of negative selection (CoNeS) score of −0.08, consistent with an AR IEI (Figure 1D) (60), and an intermediate gene damage index (GDI) of 3.6 (61). Similarly, we found no pLOF variants and only one in-frame variant of *IL23A* in the homozygous state in the general population. Overall, we found six MSMD patients from four kindreds homozygous for an experimentally proven missense LOF (eLOF) (kindred A) or pLOF (kindreds B, C, D) variant. A significant enrichment in homozygosity for such variants, which were found exclusively in patients with MSMD, was observed in the 802 MSMD patients with no identified genetic etiology relative to >3,000 patients with diverse infectious diseases from our database and the general population (*p*=5.7 × 10^−4^ and *p*=3.5 × 10^−9^, respectively). The absence of homozygous pLOF variants in the TB cohort, which includes patients without MSMD from countries in which BCG vaccination is mandatory, further suggests that AR IL-23R deficiency displays complete penetrance for BCG disease. Collectively, these findings strongly suggest that the six patients have MSMD (and CMC, for two of the patients) because of AR IL-23 deficiency.

### Two mutant alleles of *IL23R* disrupt mRNA splicing

The c.367+1G>A and c.1149–1G>A *IL23R* variants affect essential splice sites. We therefore hypothesized that they would produce aberrantly spliced transcripts. We first tested their effect on *IL23R* mRNA splicing by performing exon trapping. Both c.367+1G>A and c.1149–1G>A *IL23R* mutant mRNAs were aberrant, as shown by comparison with cells transfected with wild-type (WT) *IL23R* (Figure S1D and S1E). We then used mRNA extracted from Epstein-Barr virus-immortalized B lymphocytes (EBV-B cells) from a healthy control and P3, and from activated and expanded T cells (T-blasts) from a healthy control and P4, to generate and amplify *IL23R* cDNAs from exons 2 to 5 (healthy control and P3) or exons 8 to 11 (healthy control and P4). Sanger sequencing of the PCR products followed by TOPO-TA cloning showed a match to the canonical transcript for >90% of cDNAs from the mRNA isolated from healthy control cells, but none of those from the mRNA from patient cells (Figure S1F and S1G). Three transcripts were detected in P3’s EBV-B cells. Two of these transcripts predominated and were predicted to encode a protein with an in-frame partial deletion of the extracellular domain of IL-23R (G45_Y123delinsD, G24_Y123delinsD). The minor transcript was predicted to encode a truncated protein (G45Ffs*16) (Figure 1E). All four transcripts observed in P4 T-blasts were predicted to encode truncated proteins (Figure 1E). Thus, c.367+1 G>A and c.1149–1G>A led to aberrantly spliced and pLOF *IL23R* mRNAs in P3 and P4, respectively, at least in the cell types tested, with no leakiness. These findings suggest that these patients had AR IL-23R deficiency.

### Four loss-of-function *IL23R* alleles

**Figure 2:**
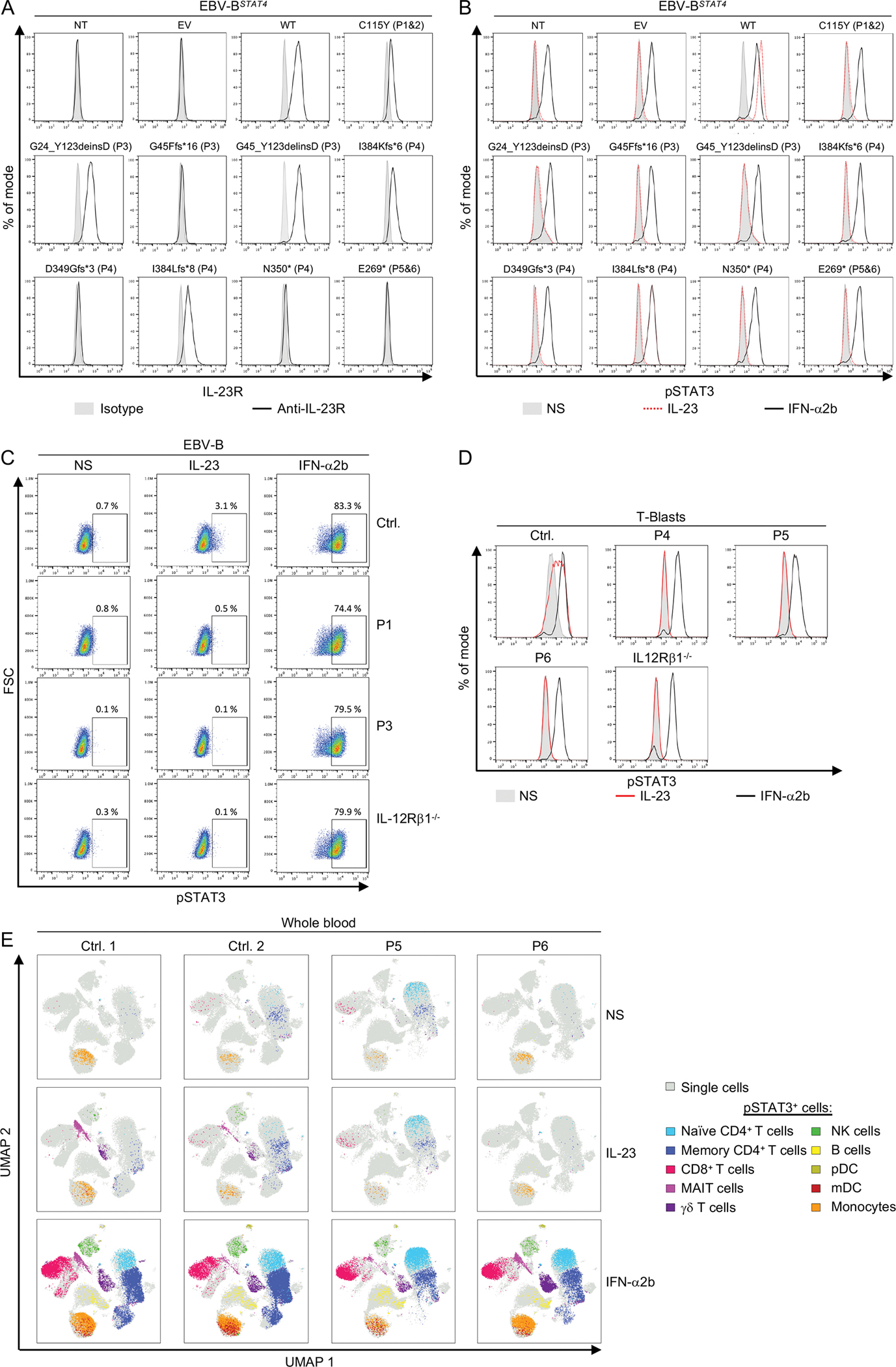
Four loss-of-function *IL23R* alleles, and six patients with AR complete *IL23R* Deficiency (**A-B**) EBV-B^*STAT4*^ cells were either left non-transduced (NT) or were transduced with lentiviruses generated with an empty vector (EV) or with vectors containing the WT or the mutated *IL23R* cDNA. Cell-surface IL-23R expression was assessed by flow cytometry (**A**). EBV-B^*STAT4*^ cells were left unstimulated (NS), or were stimulated with IL-23 or IFN-α2b as a positive control. STAT3 phosphorylation was assessed by flow cytometry (**B**). (**C**) EBV-B cells from a healthy control (Ctrl.), P1, P3 and an IL-12Rβ1-deficient patient were left unstimulated (NS), or were stimulated with IL-23 or IFN-α2b as a positive control. STAT3 phosphorylation was assessed by flow cytometry. The results shown in A-C are representative of three independent experiments. (**D**) T-blasts from a healthy control (Ctrl.), P4, P5, P6, and an IL-12Rβ1-deficient patient were left unstimulated (NS), or were stimulated with IL-23 or IFN-α2b as a positive control. STAT3 phosphorylation was assessed by flow cytometry. (**E**) Whole blood from two healthy controls, P5, and P6 was left unstimulated (NS), or was stimulated with IL-23 or IFN-α2b as a positive control. STAT3 phosphorylation in the various cell subsets was assessed by pSTAT CyTOF and the pSTAT3-positive cells were depicted in a uniform manifold approximation and projection (UMAP) visualization.

We then investigated the expression and function of the four mutant alleles. As a surrogate for these alleles, we generated cDNAs corresponding to the variants of P1/P2 (C115Y), and P5/P6 (E269*) and to the transcripts observed in the cells of P3 (G45_Y123delinsD, G24_Y123delinsD and G45Ffs*16) and P4 (I384Kfs*6, I384Lfs*8, D349Gfs*3, and N350*). We used lentivirus-mediated gene transfer to ensure the stable expression of WT or mutant *IL23R* cDNA in an EBV-B cell line that endogenously expressed STAT3 and IL-12Rβ1, but not IL-23R, and had been engineered to express STAT4 in a stable manner (referred to here as EBV-B^*STAT4*^) (11, 62). Transduction efficiency was assessed by measuring the cell-surface expression of ΔNGFR (CD271, incorporated into the backbone of the lentiviral plasmid) (Figure S2A)(63). EBV-B^*STAT4*^ cells transduced with the WT or mutant *IL23R* cDNAs produced similar amounts of *IL23R* mRNA (Figure S2B). However, relative to EBV-B^*STAT4*^ cells transduced with WT *IL23R* cDNA, EBV-B^*STAT4*^ cells transduced with mutant *IL23R* cDNA had similar (G24_Y123delinsD or G45_Y123delinsD), weaker (C115Y, I384Kfs*6, I384Lfs*8), or no (G45Ffs*16, N349Gfs*3, N350*, E269*) cell-surface expression of IL-23R (Figure 2A and S2C). We then stimulated EBV-B^*STAT4*^ cells expressing WT or mutant IL-23R proteins with IL-23, or IFN-α2b as a control. Human IL-23-dependent signaling via IL-12Rβ1/IL-23R in these cells preferentially results in STAT3 activation. EBV-B^*STAT4*^ cells expressing WT, but not mutant IL-23R phosphorylated STAT3 in response to IL-23, whereas all cells phosphorylated STAT3 in response to IFN-α2b (Figure 2B and S2D). All the *IL23R* mutant alleles were, therefore, LOF for responses to IL-23. These findings strongly suggest that the patients had AR complete IL-23R deficiency.

### Six patients with AR complete IL-23R deficiency

Our next set of experiments used patient-derived cells to capture the effects of the patients’ full genotypes at these loci in the context of their own genome. We assessed the cell-surface expression and function of IL-23R in EBV-B cells from P1 and P3. Relative to EBV-B cells from a healthy control or an IL-12Rβ1-deficient patient, cell-surface IL-23R expression was normal in P1 and much weaker in P3 (Figure S2E). These EBV-B cells were then left unstimulated or stimulated with IL-23 or IFN-α2b. EBV-B cells from the healthy control responded to IL-23 by phosphorylating STAT3, whereas EBV-B cells from P1, P3, and an IL-12Rβ1-deficient patient did not. By contrast, all cells phosphorylated STAT3 in response to IFN-α2b (Figure 2C). We then assessed the function of IL-23R in T-blasts. T-blasts from healthy controls (including P4’s sister, who is WT for IL-23R), P4, P5, and P6, and an IL-12Rβ1-deficient patient were left unstimulated, or were stimulated with IL-23 or IFN-α2b. T-blasts from healthy controls responded to IL-23 by phosphorylating STAT3, whereas T-blasts from P4, P5, P6, and the IL12Rβ1-deficient patient did not (Figure 2D and Figure S2F). T-blasts from healthy controls, P4, P5, P6, and an IL-12Rβ1 deficient patient responded to IFN-α2b by phosphorylating STAT3 or STAT4, and to IL-12 by phosphorylating STAT4, with the exception of IL-12Rβ1 deficient T-blasts (Figure 2D, and Figure S2F and S2G). Finally, whole blood from two healthy individuals, including a travel control, P5 and P6, was left unstimulated, or stimulated with IL-23 or IFN-α2b, and STAT phosphorylation was assessed by mass cytometry by time-of-flight (CyTOF) (Figure 2E and Figure S2H). In cells from healthy donors, we observed an induction of STAT3 phosphorylation, and, to a much lesser extent, an induction of STAT5 phosphorylation in response to IL-23, predominantly in mucosal-associated invariant T (MAIT) and γδ T cells, and, to a lesser extent, in natural killer (NK) and memory CD4^+^ T cells (Figure 2E and Figure S2H). In P5 and P6, no STAT phosphorylation was induced upon IL-23 stimulation in any of the leukocyte subsets tested (Figure 2E and Figure S2H). By contrast, STAT phosphorylation in response to IFN-α2b was similar in cells from P5 and P6 and in the healthy controls (Figure 2E and Figure S2H). The six patients, thus, had a complete form of AR IL-23R deficiency.

### Impaired basal IFN-γ immunity *ex vivo* in inherited IL-23R deficiency

**Figure 3:**
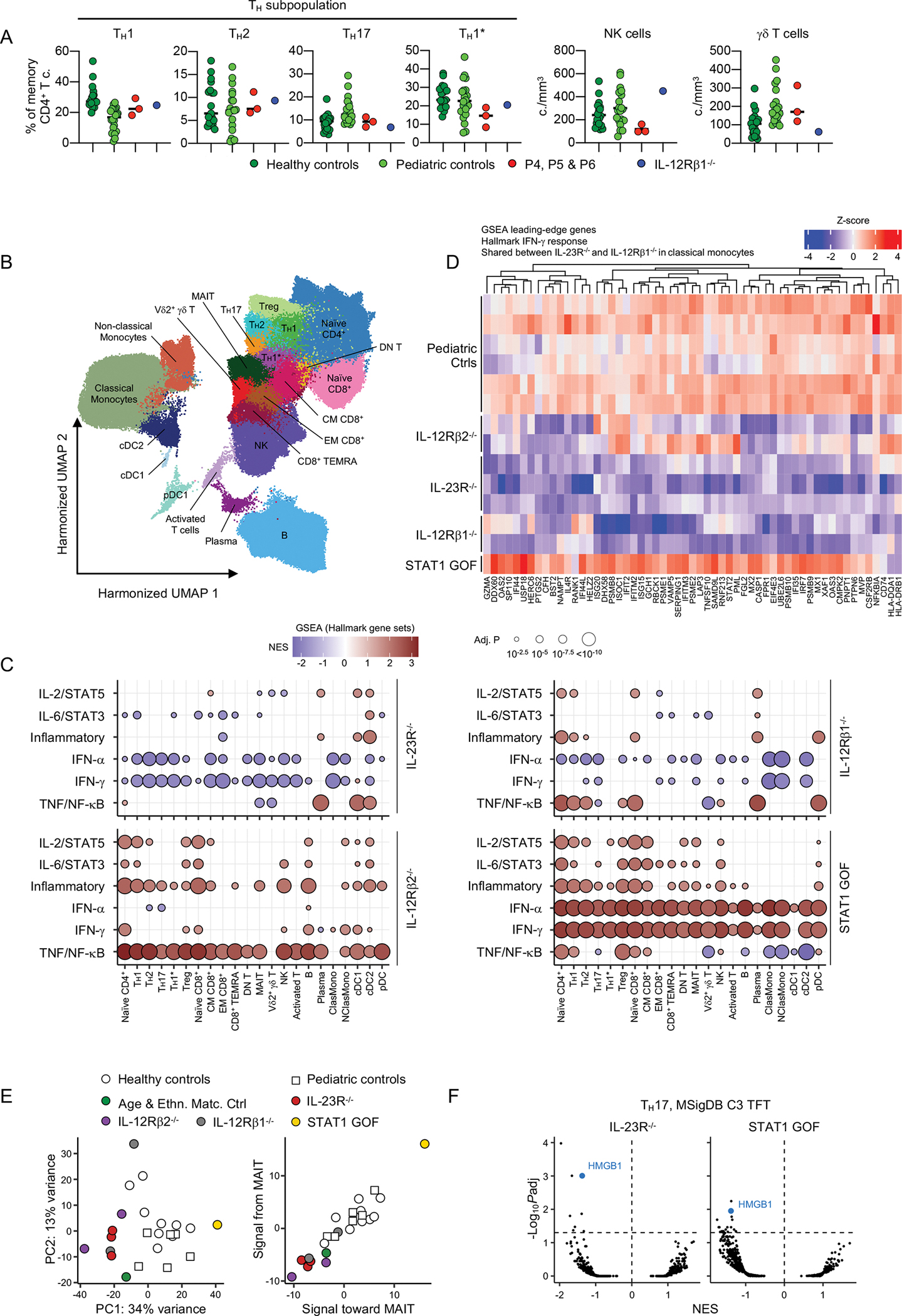
Impaired basal IFN-γ signaling *in vivo* in patients with inherited IL-23R Deficiency **(A)** Frequency of memory CD4^+^ T cells (T_H_1, T_H_2, T_H_17, T_H_1*) and absolute counts of NK and γδ T cells in 19 healthy adult controls, 20 healthy pediatric controls, and patients (P4, P5 and P6). (**B-F**) Single-cell transcriptome analysis. Cryopreserved peripheral blood mononuclear cells (PBMCs) from P3, P4 and P6 were analyzed with cryopreserved PBMCs from healthy adult and pediatric controls, two IL-12Rβ1- and two IL-12Rβ2-deficient patients, and one patient heterozygous for a STAT1 gain-of-function (GOF) variant. (**B**) Clustering analysis. After batch-correction with Harmony (28), clusters were identified manually with the aid of the SingleR pipeline (78), guided by the MonacoImmuneDataset (79). (**C**) Pseudobulk differential expression analysis. IL-23R-, IL-12Rβ1-, and IL-12Rβ2-deficient and STAT1-GOF patients were compared with healthy controls (adults and children combined). Gene set enrichment analysis (GSEA) was conducted on the fold-change ranking against the Hallmark gene sets (http://www.gseamsigdb.org/gsea/msigdb/genesets.jsp?collection=H). Only immune-related pathways are shown. (**D**) Differential gene expression in classical monocytes between adult and pediatric control cells and IL-23R-, IL12Rβ1-, and IL-12Rβ2-deficient and *STAT1*-GOF cells. Colors reflect Z-transformed normalized pseudobulk read counts. (**E**) Intercellular communication analysis with CellChat (65). Principal component analysis (PCA) of the CellChat-predicted signaling probability of communication between all cell-to-cell pairs and all receptor-ligand pairs (left panel), and PCA on CellChat-predicted probability of signaling to and from MAIT cells (right panel). (**F**) GSEA was conducted on the fold-change ranking against all transcription factor target gene sets (http://www.gsea-msigdb.org/gsea/msigdb/genesets.jsp?collection=TFT) of IL-23R-deficient and STAT1-GOF T_H_17 cells. NES, normalized enrichment score.

As a first approach to deciphering the cellular basis of MSMD and CMC in patients with inherited IL-23R deficiency, we compared the frequency of leukocyte subsets in the whole blood of healthy controls, five IL-23R-deficient patients, three IL-12Rβ1-deficient patients, and four patients heterozygous for STAT1 gain-of-function (GOF) variants (64), by CyTOF (Figure 3A and Figure S3A), and in peripheral blood mononuclear cells (PBMCs), by spectral flow cytometry (Figure S3B). The counts and frequencies of myeloid cells, NK cells, MAIT cells, γδ T cells, innate lymphoid cells (ILCs), total CD4^+^ αβ T cells, total CD8^+^ αβ T cells, and memory T_H_1, T_H_2, T_H_1*, and T_H_17 cells in IL-23R-deficient patients were within the range of healthy controls (Figure 3A and Figure S3A–S3B). We then analyzed PBMC samples from P3, P4, and P5 by single-cell RNA sequencing (scRNA-seq). We simultaneously analyzed PBMCs from two IL-12Rβ1- and two IL-12Rβ2-deficient patients, to investigate IL-23-dependent but IL-12-independent cellular phenotypes underlying MSMD, and from one STAT1 GOF patient, to investigate IL-23-dependent cellular phenotypes underlying CMC. Batch-corrected unsupervised clustering identified 22 different leukocyte subsets (Figure 3B, S3C and S3D). Pseudobulk principal component analysis (PCA) revealed the transcriptional phenotypes of different leukocyte subsets of IL-23R-, IL-12Rβ1-, and IL-12Rβ2-deficient patients, the patient with a *STAT1* GOF variant, and healthy adults and children (Figure S3E). Gene set enrichment analysis (GSEA) revealed a downregulation of genes regulated by IFN-γ in most clusters identified, except for plasma cells, type 1 conventional dendritic cells and plasmacytoid dendritic cells, in IL-23R-deficient patients relative to healthy children (Figure 3C and D). These baseline transcriptional alterations may be associated with an impairment of intercellular communication, as suggested by the probabilistic inference of intercellular communications in CellChat analysis (65) (Figure 3E). Interestingly, GSEA identified the high mobility group box 1 (HMGB1)-regulated geneset as the only transcription factor target geneset jointly downregulated in IL-23R-deficient and *STAT1* GOF T_H_17 cells (Figure 3F), suggesting that a deficiency of HMGB1 activity may underlie impaired T_H_17-dependent immunity in both IL-23R-deficient and *STAT1* GOF patients. Overall, these results suggest that IL-23R deficiency impairs IFN-γ immunity in at least 19 leukocyte subsets *in vivo*, without affecting their development *per se*.

### IL-23 induces *IFNG* in NK, MAIT and Vδ2^+^ γδ T cells

**Figure 4:**
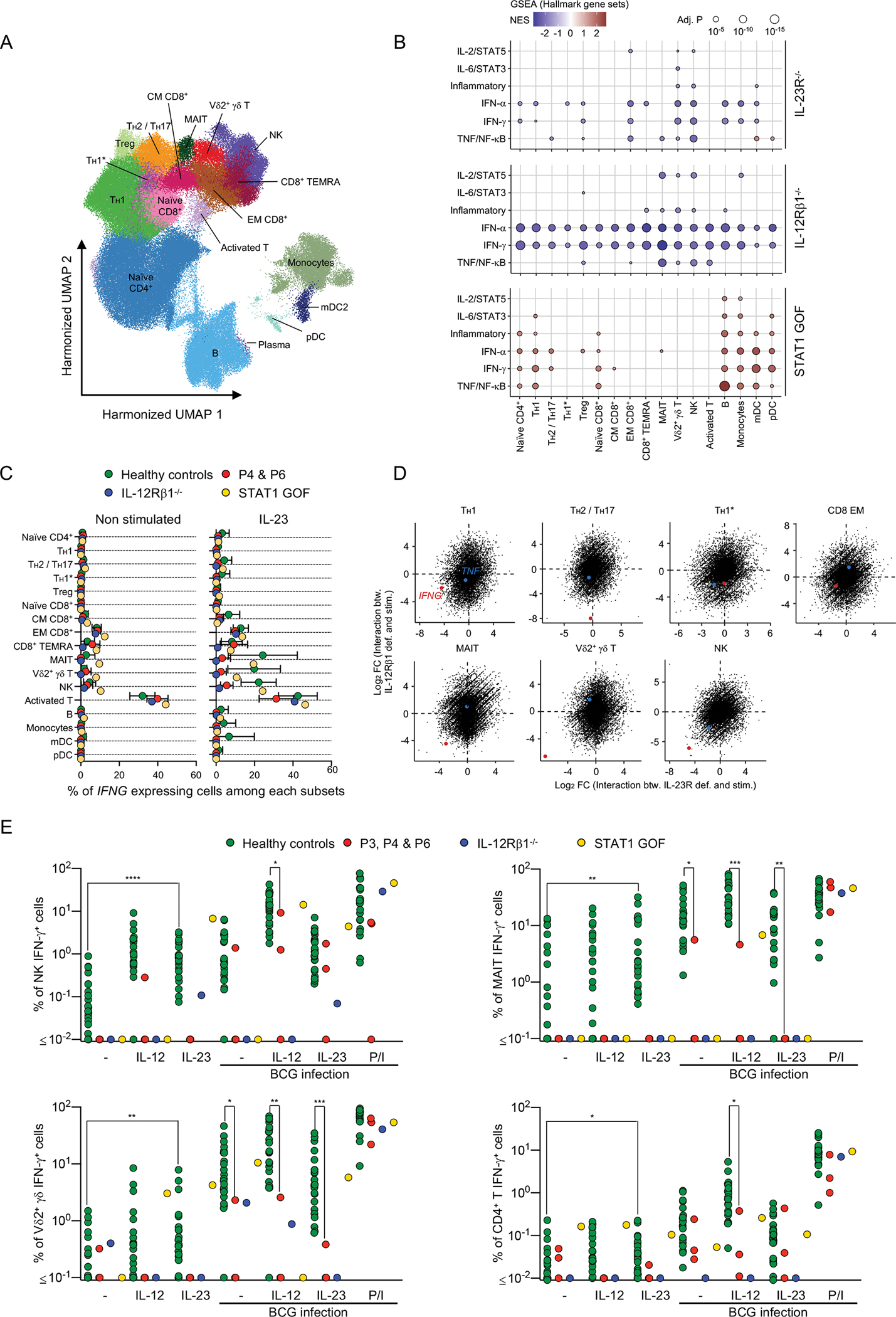
Impaired *ex vivo* IL-23-mediated production of IFN-γ in patients with inherited IL-23R deficiency (**A-D**) Single-cell RNA sequencing. We analyzed PBMCs from two IL-23R-deficient (P4 and P6), one IL-12RbR1-deficient patient, and one STAT1-GOF patient, together with six healthy controls (including P4’s sister). Cells were either left unstimulated or were stimulated with IL-23 for 6 hours. (**A**) Clustering analysis. (**B**) Pseudobulk differential expression (DE) analysis. Log_2_ fold-changes in expression were estimated with DESeq2 for the interaction between stimulation (non-stim. vs. IL-23) and genotype (WT vs. IL-23R-deficient, IL-12Rβ1-deficient or STAT1 GOF). GSEA for the Hallmark gene sets was performed on the basis of log_2_ fold-change ranking. Gene sets with FDR-adjusted *P* values below 0.05 for at least one cell subset are shown. (**C**) Percentage of cells expressing *IFNG* among each cell subset, without (left panel), or with (right panel) IL-23 stimulation. (**D**) Two-dimensional plot, log_2_ fold-change (FC) for the interaction between IL-23R deficiency and stimulation (*x* axis) and the interaction between IL-12Rβ1 deficiency and stimulation (*y* axis) in T_H_1, T_H_2/T_H_17, T_H_1*, CD8 EM, MAIT, Vδ2^+^ γδ T, and NK cells (red, *IFNG*. blue, *TNF*). (**E**) Percentages of IFN-γ^+^ cells, on intracellular flow cytometry, for the cell subsets indicated, following stimulation with and without live *M. bovis*-BCG in the presence and absence of IL-12 and IL-23, or PMA/ionomycin (P/I). Nonparametric Mann-Whitney tests were used for analysis (**E**), with *p<0.05, **p<0.01, ***p<0.001, ****p<0.0001.

We delineated IL-23-dependent cellular responses further by analyzing PBMCs from two IL-23R-deficient (P4 and P6) patients, one IL-12Rβ1-deficient patient, one STAT1 GOF patient, and six healthy individuals (including P4’s sister), by scRNA-seq, after incubation with or without IL-23 for six hours. Clustering analysis identified 18 leukocyte subsets (Figure 4A and S4A). GSEA identified IFN-γ-regulated genes with IL-23-driven induction in healthy controls, the expression of which was weaker in several leukocyte subsets in IL-23R- and IL-12Rβ1-deficient patients, including NK cells, Vδ2^+^ γδ T cells, monocytes, and myeloid dendritic cells (mDC). (Figure 4B and S4B). This observation suggested that IL-23 stimulation directly induces *IFNG* expression. Indeed, upon IL-23 stimulation, we observed an increase in the percentage of NK, MAIT, and Vδ2^+^ γδ T cells expressing *IFNG* in healthy controls and the STAT1 GOF patient, but not in IL-23R- or IL-12Rβ1-deficient patients (Figure 4C). In comparisons of IL-23R- or IL-12Rβ1-deficient patients with healthy controls, *IFNG* was the gene for which IL-23-driven induction was most strikingly impaired in Vδ2^+^ γδ T, MAIT, and NK cells (Figure 4D). By contrast, no induction of *IL17A* or *IL17F* was detected in IL-23-stimulated cells from healthy controls. Thus, human IL-23 acts predominantly as an early IFN-γ-inducing cytokine in innate-like adaptive (Vδ2^+^ γδ T, MAIT) and innate (NK) lymphocytes, which, in turn, induce IFN-γ-dependent transcriptional programs in both lymphoid and myeloid leukocyte subsets.

### Impaired *ex vivo* IL-23-mediated induction of IFN-γ in inherited IL-23R deficiency

Human IFN-γ is essential for antimycobacterial immunity, as at least 36 of the 37 known genetic etiologies of MSMD affect the production of, or response to IFN-γ. Given the IL-23-dependent induction of *IFNG* at mRNA level observed on scRNA-seq, we performed *ex vivo* experiments to test the hypothesis that inherited IL-23R deficiency impairs IFN-γ production in response to mycobacteria. We used spectral flow cytometry to assess intracellular IFN-γ production by lymphocyte subsets. Following IL-23 stimulation, the frequency of IFN-γ-expressing NK, MAIT, Vδ2^+^ γδ T, and to a lesser extent CD4^+^ T cells in healthy controls increased relative to unstimulated cells, whereas no induction of intracellular IFN-γ was observed in IL-23R-deficient cells (Figure 4E). Following infection with BCG, intracellular IFN-γ induction was mildly impaired in CD4^+^ T cells and more severely in MAIT and Vδ2^+^ γδ T cells of IL-23R-deficient patients, and this impairment was not rescued by exogenous IL-12 (Figure 4E). Intracellular IFN-γ production in response to BCG infection was not significantly impaired in the NK of the IL-23R-deficient patient (Figure 4E). These results are consistent with the IL-23-dependent induction of *IFNG* expression observed in MAIT and Vδ2^+^ γδ T cells from healthy controls on scRNAseq. Overall, IL-23R deficiency impairs IFN-γ production upon mycobacterial infection *ex vivo;* this impairment involves at least MAIT and Vδ2^+^ γδ T cells, and leads to insufficient IFN-γ-dependent protective immunity to mycobacteria.

### Normal development of BCG-specific CD4^+^ memory T cells in IL-23R-deficient patients

**Figure 5:**
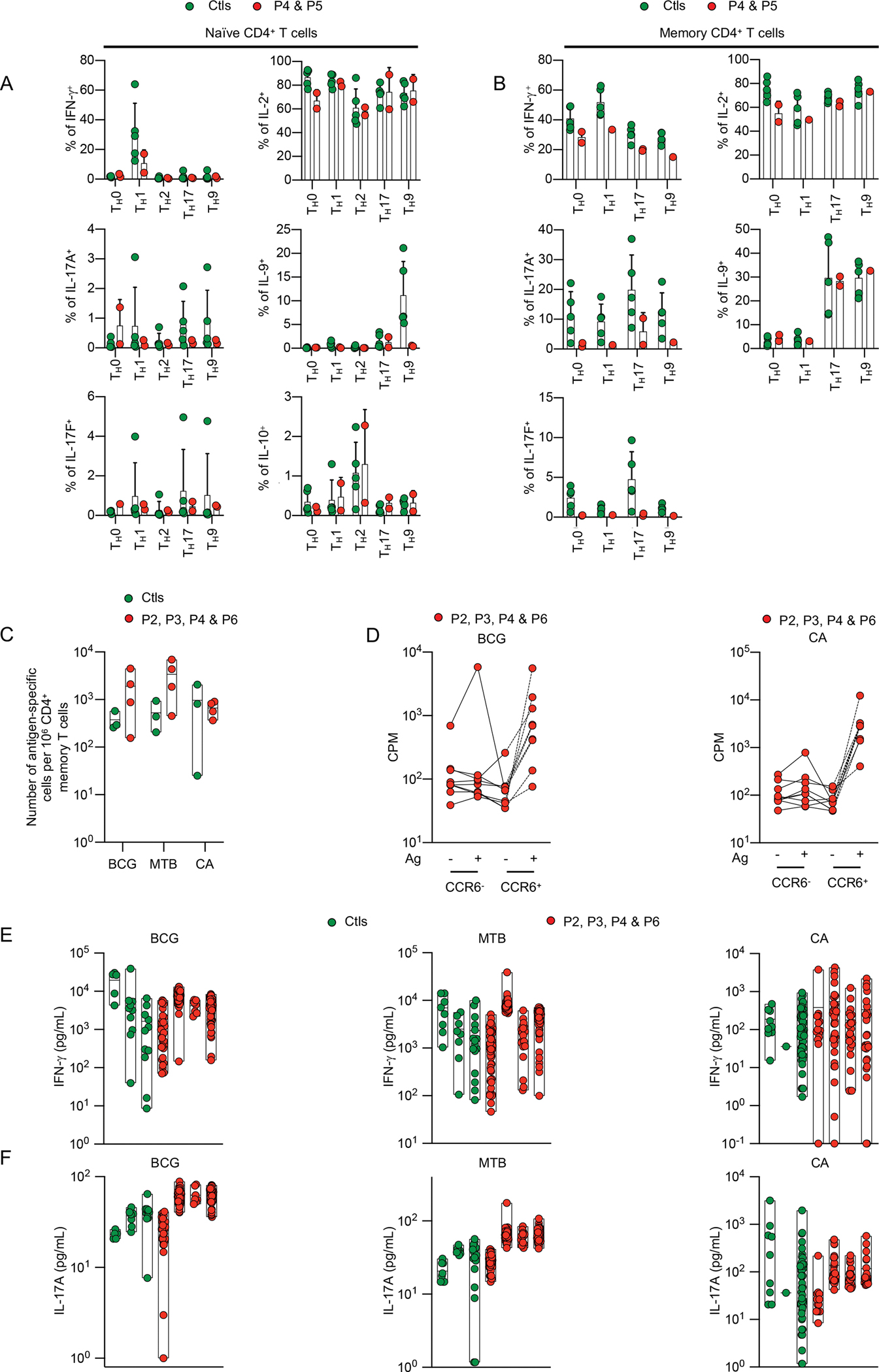
Normal development of BCG- and *C. albicans* specific memory CD4^+^ T cells in patients with inherited IL-23R deficiency (**A-B**) Naïve (**A**) or memory (**B**) CD4^+^ T cells from healthy controls (Ctls), P4, and P5 were either kept under T_H_0 (T-cell activation/expansion [TAE] beads) conditions, or were polarized under T_H_1 (TAE beads + IL-12), T_H_2 (TAE beads + IL-4), T_H_17 (TAE beads + IL-1/IL-6/IL-21/IL-23/TGF-β), or T_H_9 (TAE beads + IL-9) conditions. After five days of culture, the percentages of IFN-γ^+^, IL-17A^+^, and IL-17F^+^ cells were assessed intracellularly by flow cytometry. (**C-F**) Multiple memory CD4^+^ T cell lines were generated by stimulation with PHA, IL-2 and irradiated allogeneic PBMCs from the sorted memory CD4^+^ T cells of three healthy controls, P2, P3, P4, and P6. Lines were screened for reactivity with peptide pools covering antigens from BCG, *Mycobacterium tuberculosis* (MTB), or *C. albicans* (CA). (**C**) The proliferation of memory CD4^+^ T-cell lines after stimulation with autologous B cells pulsed with BCG, MTB, or CA peptide pools was measured by determining ^3^H-thymidine incorporation, and the frequencies (mean number per million CD4^+^ memory T cells) of BCG-, MTB-, and CA-specific memory CD4^+^ T cells were estimated assuming a Poisson distribution. (**D**) Proliferation, measured by ^3^H-thymidine incorporation, of memory CD4^+^ CCR6^−^ or CD4^+^ CCR6^+^ T-cell lines, stimulated with autologous B cells pulsed with BCG or CA peptide pools. (**E-F**) BCG, MTB, or CA reactive memory CD4+ T-cell lines from each individual were selected, and the concentrations of IFN-γ (**E**) and IL-17A (**F**) in the supernatant were determined with a Luminex assay. Each dot on the graph corresponds to the value for a single antigen-reactive T-cell line.

Our results suggested that IFN-γ induction by MAIT and Vδ2^+^ γδ T cells was deficient in IL-23R-deficient patients, but we could not rule out a contribution of impaired memory CD4^+^ T cell function to the mycobacterial disease in these patients. We therefore sorted naïve and memory CD4^+^ T cells from P4, P5, and healthy controls, and cultured them for five days under T_H_0 (T-cell activation and expansion [TAE] beads only) or T_H_1 (TAE beads plus IL-12) polarizing conditions. IFN-γ was induced in T_H_1-stimulated naïve CD4^+^ T cells from the IL-23R-deficients patients, but the amounts in which it was produced and secreted (assessed by determining the percentage of IFN-γ^+^ cells and the concentration of IFN-γ in the supernatant) were at or below the lower end of the range for naïve CD4^+^ T cells from healthy donors (Figure 5A and S5A). Similar results were obtained for IL23R-deficient memory CD4^+^ T cells in that these cells were able to produce and secrete IFN-γ, but in smaller amounts than CD4^+^ T cells from healthy donors (Figure 5B and S5B). We then assessed the impact of IL-23R-deficiency on the proportion of BCG-reactive memory CD4^+^ T cells and their IFN-γ production. Following stimulation with tuberculin purified protein derivative (PPD), the proportion of IFN-γ^+^ cells among CD40L^+^/CD69^+^ reactive memory CD4^+^ T cells was similar between healthy controls and the IL-23R-deficient patients (Figure S5C). Following polyclonal stimulation of T cells with antibody complexes binding CD2, CD3, and CD28, the proportion of CD40L^+^/CD69^+^ activated memory CD4^+^ T cells, including that of IFN-γ^+^ cells, was lower in IL-23R-deficient patients, when compared with healthy controls (Figure S5C). Finally, we assessed the reactivity to BCG of memory CD4^+^ T cells expanded *in vitro* with PHA, IL-2 and irradiated allogeneic PBMCs; the frequency of BCG-specific memory CD4^+^ T cells was similar between healthy controls and IL-23R-deficient patients (Figure 5C and Figure S5D and S5E). Most of these cells belonged to the CCR6^+^ compartment (Figure 5D and Figure S5F), suggesting that IL-23R-deficiency does not impair the development of BCG-specific T_H_1* cells (66). IFN-γ production by BCG-specific memory CD4^+^ T cells was also similar between IL-23R-deficient patients and healthy controls (Figure 5E). Overall, IL-23R deficiency slightly decreased the capacity of CD4^+^ T cells to differentiate into IFN-γ-producing cells, but did not appear to affect the development of BCG-specific memory CD4^+^ T cells. The normal frequency of BCG-specific CD4^+^ memory T cells and their intact ability to produce IFN-γ relative to healthy individuals may account for the absence of mycobacterial disease recurrence in the seven IL-23R-deficient patients described to date (11, 67) (see Table S1).

### Impaired *ex vivo* IL-23-mediated production of IL-17 cytokines in inherited IL-23R deficiency

**Figure 6:**
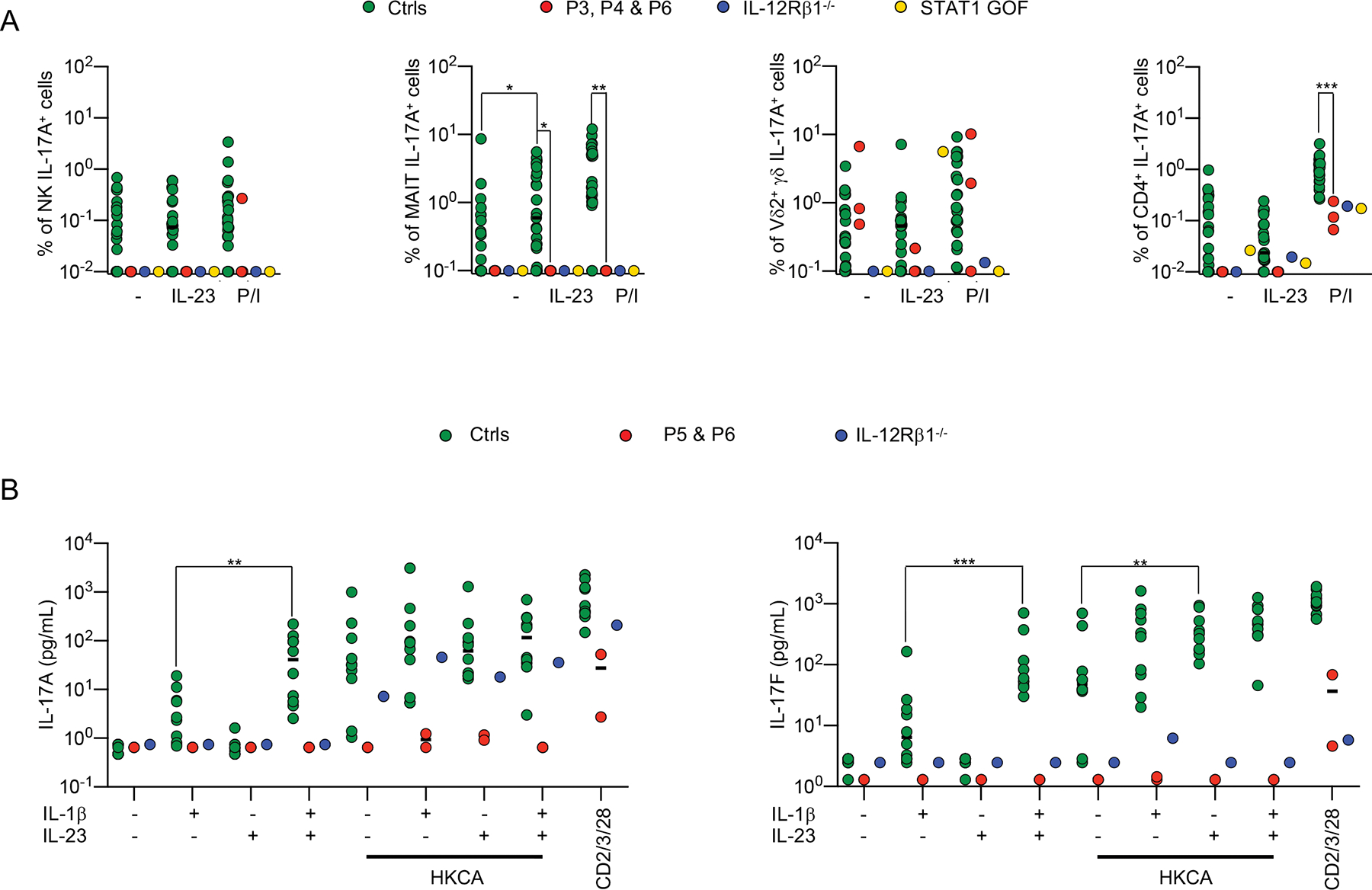
Impaired *ex vivo* IL-23-mediated production of IL-17 cytokines in patients with inherited IL-23R deficiency (**A**) Percentages of IL-17A^+^ cells on intracellular flow cytometry for the indicated cell subsets, from the indicated individuals (healthy controls, Ctrls; patients, P3, P4, P6; one IL-12Rβ1 deficient patient, and one STAT1-GOF patient) unstimulated (–), or stimulated with IL-23 or PMA/ionomycin (P/I). (**B**) IL-17A and IL-17F secretion by PBMCs, assessed by Legendplex assays on the supernatants, for the indicated individuals (healthy controls, Ctrls; patients P5, P6; and one IL-12Rβ1-deficient patient). The cells were cultured for five days, and were then either left unstimulated (–), or stimulated with IL-1β (+), and/or IL-23 (+), in the presence (+) or absence (–) of heat-killed *C. albicans* (HKCA), or anti-CD2/CD3/CD28 mAb-coated beads (CD2/3/28). Nonparametric Mann-Whitney tests were used for analysis in panels **A-B** (*p<0.05, **p<0.01, ****p<0.0001).

IL-17A/F are cytokines essential for anti-*Candida* mucosal immunity, as all 14 known genetic etiologies of CMC (isolated or syndromic) affect IL-17-mediated immunity (36, 68). We therefore performed *ex vivo* experiments to assess the impact of inherited IL-23R deficiency on IL-17-dependent immunity to *Candida*. We first assessed the responses of PBMCs from P3, P4, and P5 to *ex vivo* stimulation for eight hours with phorbol 12-myristate 13-acetate and ionomycin (P/I). We measured intracellular IL-17A production in different lymphocyte subsets by spectral flow cytometry. Following P/I stimulation, the frequencies of IL-17A^+^ MAIT and CD4^+^ T cells were significantly lower in IL-23R-deficient patients than in healthy controls (Figure 6A). We assessed the response of IL-23R-deficient PBMCs to *ex vivo* stimulation with IL-23 for 48 hours. We observed no increase in the frequency of IL-17A^+^ MAIT cells upon IL-23 stimulation in IL-23R-deficient patients, contrary to our findings for healthy controls (Figure 6A). Frequencies of IL-17A^+^ Vδ2^+^ γδ T, NK, and CD4^+^, cells did not increase upon IL-23 stimulation in healthy controls. We then assessed the responses of IL-23R-deficient PBMCs to *ex vivo* stimulation with heat-killed *C. albicans* (HKCA). HKCA stimulation induced the secretion of IL-17A/F and IL-22 in healthy control PBMCs, but not in PBMCs from P3, P4, P5, and P6 (Figure 6B and Figures S6A and B). Finally, we stimulated PBMCs from healthy individuals and IL-23R-deficient patients with IL-23, with or without IL-1β, in the presence or absence of HKCA. We found that the stimulation of control PBMCs with IL-23 in the presence of IL-1β enhanced the production of IL-17A, IL-17F and IL-22 relative to stimulation with IL-1β or IL-23 alone, and that IL-23 further induced IL-17F production in the presence of HKCA. By contrast, under the same stimulation conditions, no IL-17A, IL-17F, or IL-22 was detected in the supernatant of PBMCs from IL-23R-deficient patients (Figure 6B and Figure S6B). Overall, MAIT cells from IL-23R-deficient patients, like those of IL-12Rβ1-deficient patients or STAT1 GOF patients, displayed impaired IL-17A induction upon IL-23 stimulation, and PBMCs from IL-23R- and IL-12Rβ1-deficient patients displayed impaired IL-17A/F production in response to heat-killed *C. albicans*, possibly accounting for their risk of CMC (41–47).

### Normal development of *C. albicans*-specific CD4^+^ memory T cells in IL-23R-deficient patients

CMC displays incomplete penetrance in IL-23R deficiency, suggesting that IL-17 production may be preserved in some cell subsets. We therefore studied the impact of IL-23R deficiency on the development of T_H_17 cells. We sorted naïve and memory CD4^+^ T cells from P4, P5, and healthy individuals. After five days of culture under T_H_17 polarizing (TAE beads plus TGF-β, IL-1β, IL-6, IL-21 and IL-23) conditions, the production of IL-17A and IL-17F (assessed by determining the percentage of IL-17A^+^ and IL-17F^+^ cells among naïve and memory CD4^+^ T cells, and the concentration of IL-17A and IL-17F secreted by naïve and memory CD4^+^ T cells) by IL-23R-deficient cells was impaired relative to that of healthy controls, but not entirely abolished (Figure 5A and B, and Figure S5A and B). We then assessed the reactivity to *C. albicans* of *in vitro*-expanded CD4^+^ memory T cells from P2, P3, P4, P6 and healthy controls; the frequency of *C. albicans*-specific cells was similar for healthy controls and IL-23R-deficient patients (Figure 5C and Figure S5D). Most of these cells belonged to the CCR6^+^ compartment (Figure 5D), suggesting that IL-23R-deficiency does not impair the development of *C. albicans*-specific T_H_17 cells (66). Moreover, IL-17A production by *C. albicans*-specific CD4^+^ memory T cells was similar for IL-23R-deficient patients and healthy controls (Figure 5F). Overall, these results suggest that IL-23R deficiency slightly decreases the capacity of CD4^+^ T cells to differentiate into T_H_17-producing cells but does not impair the development of *C. albicans*-specific CD4^+^ memory T cells. The normal frequency of *C. albicans*-specific CD4^+^ memory T cells and the conservation of their capacity to produce IL-17A may be sufficient to protect against CMC, in some patients, potentially accounting for the incomplete penetrance of CMC in IL-23R-deficient patients (36, 41–44).

## DISCUSSION

We report the characterization of six patients from four unrelated Iranian kindreds with AR complete IL-23R deficiency. These patients have normal numbers of circulating leukocytes from various subsets, including cell types that normally express IL-23R, such as NK, iNKT, MAIT, Vδ2^+^ γδ T, T_H_1, T_H_17 and T_H_1* cells (11, 69). The vulnerability of some IL-12p40-, IL-12Rβ1-, and IL-23R-deficient patients to CMC suggests that human IL-23 plays a crucial role in IL-17A/F-mediated defense against mucocutaneous infections with *Candida* spp. in a subset of affected individuals. Indeed, CMC is seen in ~ 25 to 35% of patients with any of these three disorders, implying that IL-23 is redundant for defense against *Candida* in most individuals. We observed an IL-23-driven induction of IL-17A *ex vivo* only for MAIT cells from healthy individuals, this induction being abolished in IL23R-deficient patients. We also found impaired IL-17A/F secretion by IL-23R-deficient leukocytes in response heat-killed *C. albicans*, possibly due to the impaired IL-23 responses of MAIT cells. In a mouse model of experimental autoimmune encephalomyelitis (EAE), IL-23 drives the expansion of the IL-17A^+^ CD4^+^ T-cell population (70). IL-23-deficient mice (*Il23p19*^*−/−*^) have a higher *C. albicans* burden than WT mice in models of oropharyngeal candidiasis (OPC) and cutaneous *C. albicans* infection (71, 72). The molecular and cellular basis of CMC in patients with IL-23R deficiency is probably similar to that in IL-12p40- and IL-12Rβ1-deficient patients, whose leukocytes also display no IL-23 signaling. In patients with any of these three disorders, CMC displays incomplete penetrance, which does not seem to be explained by the interindividual variability of IL-17A/F production by leukocyte subsets. Incomplete penetrance may reflect the observations that the development of *C. albicans*-specific memory CD4^+^ T cells is preserved in IL-23R-deficient patients, and that IL-17A induction is controlled by IL-23 in only one lymphocyte subset (MAIT cells), at least *ex vivo*.

By contrast, human IL-23/IL-23R signaling was surprisingly found to be essential for IFN-γ-mediated immunity to mycobacteria in all IL-23R-deficient patients identified. Our previous report of kindred A in 2018 was suggestive, but not conclusive, as only two siblings with MSMD were reported, and the cellular basis of the disease was not characterized (11). The data reported here, together with the recent description of disseminated environmental mycobacterial disease in an IL-23R-deficient patient (67), further suggest that human IL-23 is essential for host defense against even weakly virulent mycobacteria, as IL-23R deficiency underlies MSMD with complete penetrance in five unrelated kindreds with different and private *IL23R* genotypes. An ascertainment bias is unlikely to have affected this assessment, as two of the six MSMD patients are siblings of index cases. We also deciphered the cellular basis of mycobacterial disease. We showed, by scRNAseq, that inherited IL-23R deficiency impairs IFN-γ-dependent signaling in MAIT, Vδ2^+^ γδ T, T_H_1, T_H_17, and T_H_1* cells in the basal state *in vivo*. We speculate that various environmental stimuli continuously trigger low-level IFN-γ signaling in an IL-23-dependent manner in the basal state in healthy individuals. This model is further supported by the observations that IL-23R-deficient MAIT and Vδ2^+^ γδ cells display impaired induction of *IFNG* mRNA and IFN-γ protein upon stimulation with IL-23 and live BCG mycobacteria *ex vivo*. However, these findings do not exclude the possible contribution of other cellular subsets to IL-23-dependent immunity to mycobacteria, such as IL-23R-deficient CD4^+^ T cells, which showed decreased production of IFN-γ under some conditions. Exogenous IL-12 did not completely rescue the defective IFN-γ production of IL-23R-deficient cells, indicating that IL-12 and IL-23 control IFN-γ production in different ways. Consistently, we previously showed that a partial and selective defect of IL-23 signaling due to a common homozygous missense variant of *TYK2* (P1104A) predisposes patients to TB (9, 12). The physiological significance of this finding is supported by its evolutionary impact; the frequency of the P1104A *TYK2* allele has decreased in Europeans over the last 2,000 years, probably due to the negative selection exerted by TB (and possibly, to a lesser extent, by other intramacrophagic pathogens) (73). In this context, our report further attests to the essential contribution of human IL-23 to antimycobacterial immunity.

Our findings therefore suggest that human IL-12 and IL-23 are functionally more closely related, via their induction of IFN-γ, than previously thought based on the classical T_H_1/T_H_17 paradigm established in mice (49–51, 74). The discovery of IL-23 and IL-23R in the early 2000s was associated with the definition of T_H_17 cells as a distinct T-cell lineage, different from T_H_1 and T_H_2 cells (49–51, 74). In this view, IL-12 is an IFN-γ-inducing signature cytokine, whereas IL-23 is an IL-17-inducing signature cytokine (49–51). The observation that the administration of IFN-γ — the archetypal T_H_1 cytokine — protects mice from EAE, whereas *Ifng* deletion worsens EAE suggested that the T_H_1/T_H_2 dichotomy was insufficient (50, 74). Subsequent findings that IL-23 promotes the expansion of a T-cell population that produces IL-17, IL-6, and TNF (70), and EAE resistance in IL-23-deficient mice paved the way for the definition of T_H_17 cells (75). This led to the IL-12/IFN-γ-T_H_1 and IL-23/IL-17-T_H_17 dichotomy (50, 51). The experiment of Nature reported here suggests that this dichotomy holds only partially in humans, and that human IL-23 is instead primarily an antimycobacterial IFN-γ-inducing cytokine. IL-12 is essential for IFN-γ production by antigen-specific CD4^+^ αβ T cells (66), which are purely adaptive T cells, but IL-23 is essential for IFN-γ production primarily by innate-like adaptive cells (MAIT and Vδ2^+^ γδ T). We, thus, speculate that IL-23 may also be the phylogenetically ancestral IFN-γ-inducing cytokine. Our findings further suggest that the mechanisms underlying the therapeutic efficacy of IL-23p19 blockers may involve an inhibition not only of excessive IL-17 immunity, if indeed this immunity is inhibited at all, but also, and perhaps predominantly or exclusively, of excessive IFN-γ immunity (51, 74). They also challenge the involvement of IL-17 immunity inferred from IL-23R signals detected in genome-wide association studies (51, 76, 77).

## MATERIALS AND METHODS

### Study design

We performed WES on a cohort of 802 patients with MSMD of unidentified genetic etiology. We identified six patients homozygous for one out of four different eLOF or pLOF *IL23R* variants. All six patients had a history of MSMD and two suffered from CMC. We examined the consequences of these mutations both in isogenic overexpression experiments and in the patients’ cells. We found that the four *IL23R* variants abolish cell response to IL-23. We performed *ex vivo* and *in vitro* experiments using PBMCs from the patients and controls, to investigate the impact of IL-23R deficiency on IFN-γ-dependent immunity to mycobacteria and IL-17-dependent immunity to *C. albicans*. Conclusions were drawn from analyzing the results from aforementioned approaches collectively.

## Supplementary Material

Supplementary Materials and Methods Supplementary Figure 1: Private homozygous *IL23R* variants in four Iranian kindreds
Supplementary Figure 2: Loss-of-function IL23R alleles and AR complete IL23R deficiency
Supplementary Figure 3: Development of peripheral mononuclear hematopoietic cells in IL-23R-deficient patients
Supplementary Figure 4: Impaired *ex vivo* IL-23-mediated production of IFN-γ in cells from patients with IL-23R deficiency
Supplementary Figure 5: Normal development of BCG- and *C. albicans*-specific memory CD4^+^ T cells in patients with inherited IL-23R deficiency
Supplementary Figure 6: Impaired *ex vivo* IL-23-mediated production of IL-17 cytokines in patients with inherited IL-23R deficiency
Table S1: Summary of the medical history of patients with inherited IL-23R deficiency
Table S2: List of all homozygous coding, essential-splicing site and splice-site variants with a CADD above the MSC not present in the homozygous state in GnomAD but detected in the analyses of the exomes of P1 to P6
Table S3: Genotypes of IL-12Rβ1- and IL-12Rb2-deficient patients and STAT1-GOF patients included as controls
Table S4: Gating strategy for the assessment of STAT phosphorylation by mass cytometry (CyTOF).
Table S5: Gating strategy for deep immunophenotyping by mass cytometry (CyTOF).
Table S6: Gating strategy for deep immunophenotyping by spectral flow cytometry.
Table S7: Gating strategy for the *ex vivo* evaluation of IFN-γ^+^ and IL-17A^+^ cells after PBMC stimulation with IL-23 or IL-12, in the presence or absence of BCG infection.
Table S8: Gating strategy for the *ex vivo* evaluation of BCG-reactive memory CD4^+^ T cells.
Table S9 : Sequence of primers used for this study

## Data Availability

All raw and processed data and biological materials, including immortalized cell lines from patients, are available upon request from the corresponding authors under a material/data transfer agreement.
