# Supplementary material for: Human IL-23 is essential for IFN-γ-dependent immunity to mycobacteria
Source: Sci Immunol. Author manuscript; Available in PMC 2023 Apr 3. (PMC10069949; doi:10.1126/sciimmunol.abq5204)
Supplement: Supplementary Materials and Methods — Supplementary Figure 1: Private homozygous IL23R variants in four Iranian kindreds Supplementary Figure 2: Loss-of-function IL23R alleles and AR complete IL23R deficiency Supplementary Figure 3: Development of peripheral mononuclear hematopoietic cells in IL-23R-deficient patients Supplementary Figure 4: Impaired ex vivo IL-23-mediated production of IFN-γ in cells from patients with IL-23R deficiency Supplementary Figure 5: Normal development of BCG- and C. albicans-specific memory CD4+ T cells in patients with inherited IL-23R deficiency Supplementary Figure 6: Impaired ex vivo IL-23-mediated production of IL-17 cytokines in patients with inherited IL-23R deficiency Table S1: Summary of the medical history of patients with inherited IL-23R deficiency Table S2: List of all homozygous coding, essential-splicing site and splice-site variants with a CADD above the MSC not present in the homozygous state in GnomAD but detected in the analyses of the exomes of P1 to P6 Table S3: Genotypes of IL-12Rβ1- and IL-12Rb2-deficient patients and STAT1-GOF patients included as controls Table S4: Gating strategy for the assessment of STAT phosphorylation by mass cytometry (CyTOF). Table S5: Gating strategy for deep immunophenotyping by mass cytometry (CyTOF). Table S6: Gating strategy for deep immunophenotyping by spectral flow cytometry. Table S7: Gating strategy for the ex vivo evaluation of IFN-γ+ and IL-17A+ cells after PBMC stimulation with IL-23 or IL-12, in the presence or absence of BCG infection. Table S8: Gating strategy for the ex vivo evaluation of BCG-reactive memory CD4+ T cells. Table S9 : Sequence of primers used for this study [file NIHMS1878751-supplement-Supplementary_Materials_and_Methods.pdf]

SUPPLEMENTARY MATERIALS AND METHODS

Patients

Informed consent for participation in this study was obtained in accordance with local regulations, with approval from the institutional review board (IRB). The study was approved by the institutional ethics committees of The Rockefeller University and Necker Hospital for Sick Children, and was performed in accordance with the requirements of these bodies. The experiments described here were performed in Australia, France, Qatar, Switzerland and the United States of America, in accordance with local regulations, and with the approval of the IRBs of the corresponding institutions.

WES, variant filtering and Sanger sequencing

Genomic DNA was extracted from whole-blood samples from the patients and their relatives. WES was performed, and homozygosity rate was estimated from the patients' genomic DNA, as previously described (*10*). Minor allele frequencies (MAFs) in the general population, as reported in gnomAD database v2.1.1, and precomputed combined annotation–dependent depletion (CADD) scores (v1.6) were used for variant filtering. The mutation significance cutoff (MSC) was calculated as previously described (*55*). For the verification of variants, exons and flanking regions were amplified from DNA with DreamTaq DNA polymerase. They were then sequenced by the Sanger method with the Big Dye Terminator v3.1 kit (Thermo Fisher Scientific), and subjected to capillary electrophoresis (#A30469, Applied Biosystems 3500xL system, Thermo Fisher Scientific).

Enrichment analysis

An analysis of enrichment in pLOF variants of *IL23R* was conducted on the WES data of 802 patients with MSMD of unidentified genetic etiology from our in-house exome database.

As controls, we used 3410 individuals of diverse ethnic origins with various non-mycobacterial or fungal infections from our in-house database. We used the Firth penalized logistic regression method (*80*), as implemented in the R (v4.1) logistf package (*81*), to compare the proportion of individuals with pLOF variants between the MSMD and control groups.

The analyses were adjusted for the first five principal components (PCs) to account for the ethnic heterogeneity of the samples. The principal component analysis was performed with Plink v1.9 software on whole-exome sequencing data for 32,733 exonic variants with a MAF > 1% and a call rate > 99%.

Phage immunoprecipitation sequencing (PhIP-Seq)

The VirScan phage library used for PhIP-Seq in this study has been described elsewhere (*82*). In brief, we used an expanded version (*83*) of the original VirScan phage library described by Xu et. al. (*84*). Custom sequencing libraries were prepared as previously described (*85*), and sequencing was performed with the NextSeq system (Illumina). Filtering for significantly enriched peptides was performed by imputing -log10(P-values) by fitting a zero-inflated generalized Poisson model to the distribution of output counts and regressing the parameters for each peptide sequence based on the input read count. Peptides with a reproducibility threshold exceeding 2.3 [-log10(P-value)] for two technical sample replicates were considered to display significant enrichment. We then calculated microbial score values as described by Xu et al. (*84*), by counting the number of non-homologous, peptides displaying significant enrichment per species (i.e., we counted the peptides enriched in each microbial species without linear sequence identity over seven or more amino acids, the estimated size of a linear B-cell epitope). Finally, the scores were adjusted by dividing by established species-specific significance cutoff values (*86*). The adjusted species scores served as a measurement of the breadth of the antibody repertoire (i.e., reflecting the diversity of antibodies) against a

pathogen. Samples with an adjusted species score ≥ 1 were considered to be seropositive for the corresponding microbial species.

Exon trapping assay

DNA segments encompassing the *IL23R* exon 3 region (Chr1: 67634951 to Chr1:67635672 region, GRCh37 reference) and the exon 10 region (Chr1: 67720954 to Chr1: 67721800 region, GRCh37 reference) were amplified from genomic DNA extracted from whole-blood samples from P3 and P4, respectively, and from a healthy control, and were inserted between the *Xho*I and *Bam*HI sites of the pSPL3 vector. Plasmids containing wild-type (WT) and mutant *IL23R* exon 3 or 10 regions were then used to transfect COS-7 cells. After 24 hours, total RNA was extracted and reverse-transcribed. The cDNA products were amplified, with primers binding to the flanking HIV-TAT sequences of the pSPL3 vector, and ligated into the pCR™4-TOPO® vector (Invitrogen). Stellar™ cells (Takara) were transformed with the resulting plasmids. Colony PCR and sequencing with primers binding to the flanking HIV-TAT sequences of pSPL3 were performed to assess the splicing products transcribed from the WT and mutant alleles.

TOPO-TA cloning

Total RNA was extracted from the EBV-B cells of P3 and a healthy control, and the T-blasts of P4 and a healthy control, with the Quick-RNA Microprep kit (Zymo). The RNA was reverse-transcribed to generate cDNA with SuperScript II (Invitrogen). Specific primers binding to exon 2 (forward primer, ATGAATCAGGTCACTATTCAATGGG) and exon 5 (reverse primer, CAAGTACTTCTTGCCACCTTGTAATG), or exon 8 (forward primer, GGGCTAACAGTTGCTTCCATC) and exon 11 (reverse primer, CCTGAGTCTAAGGAATCAACTGGTG) were used to amplify *IL23R* cDNA by PCR. The

PCR products were purified and ligated into a pCR™4-TOPO® vector (Invitrogen). Stellar™ cells (Takara) were transformed with the resulting plasmids. Colony PCR and sequencing with the primers used to amplify the *IL23R* cDNA were performed to assess the splicing products transcribed from the WT and mutated alleles.

Cell culture

PBMCs were isolated by Ficoll-Hypaque density centrifugation (Amersham-Pharmacia-Biotech). EBV-B cells were cultured in RPMI-1640 medium supplemented with 10% fetal calf serum (FCS). For T-blast induction, PBMCs were cultured in ImmunoCult™ - XF T Cell Expansion Medium (Stemcell) in the presence of ImmunoCult™ Human CD3/CD28/CD2 T-cell activator (12.5 □L.mL^{-1}) and human recombinant IL-2 (100 ng/mL, Novartis).

Lentivirus production and transduction

Plasmids containing the WT human *IL23R* (Origene #RG211477) ORF were obtained, the tag was removed, and the variants studied here were generated by site-directed mutagenesis, with specific primers and the CloneAmp HiFi PCR premix (Takara). These ORFs were introduced into the pTRIP-SFFV-□NGFR-2A plasmid and used to generate lentiviral particles. HEK293T cells were transfected with pCMV-VSV-G, pHXB2-env, psPAX2, pTRIP-SFFV-ΔNGFR-2A (empty vector, EV), or the same vector backbone containing one of the *IL23R* ORFs, in the presence of X-tremeGENE HP (Sigma Aldrich). The medium was replaced after 8 hours of incubation. On day 0, 24 hours after the HEK293T cell medium was changed, the viral supernatant was recovered and passed through a filter with 0.2 µm pores. Protamine sulfate (8 µg/mL) was added to the viral supernatant, which was then added to the EBV-B^{STAT4} cells (immediately after seeding). The cells were then spinoculated for 2 hours at 1200 x *g* and

25°C. The cells were cultured at 37°C under an atmosphere containing 5% CO_2. The transduced cells were purified by MACS, with a magnetic bead-conjugated anti-NGFR antibody (Miltenyi Biotec), according to the manufacturer's protocol, and flow cytometry confirmed purity to be > 80%.

RNA analysis and RT-qPCR

Total RNA was extracted with the Quick-RNA Microprep kit (Zymo), and reverse-transcribed to generate cDNA with the High-Capacity RNA-to-cDNA™ kit (Applied Biosystems™). Quantitative PCR was then performed on the RNA with the Applied Biosystems probes/primers specific for *IL23R*-FAM (Hs00332759_m1) and β-glucuronidase-VIC (4326320E), for normalization. Results are expressed according to the ΔCt method.

Assessment of cell-surface IL-23R expression, STAT3 and STAT4 phosphorylation by flow cytometry

For measurement of the cell-surface expression of IL-23R, we plated EBV-B^{STAT4} and EBV-B cells in 96-well plates, at a density of 1×10^6 cells per well. The cells were stained with either biotinylated goat anti-IL23R (R&D, Cat: BAF 1400, 1:50) or biotinylated goat control isotype (R&D, Cat: BAF 106, 1:50) antibody, and the Aqua Dead cell marker (Thermo Fisher Scientific). Cells were then washed and incubated with PE-conjugated streptavidin (BD Pharmingen™). Levels of pSTAT3 or pSTAT4 in EBV-B^{STAT4}, EBV-B cells and T-blasts were determined in serum-starved cells. Cells (1×10^6) were stained with the Aqua Dead cell viability marker (Thermo Fisher Scientific) before being incubated for 30 minutes with IL-23 (100 ng/mL, R&D), IL-12 (50 ng/mL, R&D), or IFN-α2b (10^5 IU/mL, Intron A, Merk). Cells were then incubated at 37°C in Fix buffer I (1:1 volume, BD Biosciences), permeabilized by incubation at room temperature in Perm buffer III (BD Biosciences) and stained at 4°C with

PE-conjugated anti-STAT3-pY705 antibody (BD Biosciences, Cat: 612569, Clone: 4/P-STAT3, 1:25), or with PE-conjugated anti-STAT4-pY693 antibody (BD Biosciences, Cat: 562073, 1:25). The cells were analyzed on a Gallios flow cytometer (Beckman Coulter), and the results were analyzed with FlowJo software.

Assessment of STAT phosphorylation by mass cytometry (CyTOF)

Fresh blood from two healthy individuals (including a travel control), and two IL-23R-deficient patients (P5 and P6) was either left unstimulated, or was stimulated with IL-23 (100 ng/mL, R&D), or IFN-α2b (10^5 IU/mL, Intron A, Merk). After 20 minutes of incubation, samples were fixed and frozen in protein stabilizer (PROT1, Smart Tube Inc.). Stabilized blood samples were thawed according to the manufacturer's recommended protocol and washed with barcode permeabilization buffer (Fluidigm). Samples were uniquely barcoded with the Cell-ID 20-Plex Pd Barcoding Kit (Fluidigm), washed and pooled. An Fc-block and a heparin-block were used to prevent non-specific binding. Cells were then incubated with an antibody cocktail for surface markers to identify major immune populations. All the antibodies were purchased pre-conjugated or were conjugated in-house with X8 MaxPar conjugation kits (Fluidigm). After surface staining, the samples were washed, permeabilized in methanol, stored at -80°C overnight, washed again, blocked with heparin and stained with a cocktail of antibodies against intracellular targets, including markers of phosphorylation. The cells were washed again and incubated in freshly diluted 2.4% formaldehyde containing 125 nM Ir Intercalator (Fluidigm), 0.02% saponin and 30 nM OsO_4 (ACROS Organics) for 30 minutes at room temperature. Samples were then washed with PBS + 0.2% BSA, PBS, and then CAS buffer (Fluidigm) for immediate acquisition. The final suspension in CAS buffer contained one million cells per mL and a 1/20 dilution of EQ beads (Fluidigm). Following routine instrument optimization, samples were acquired at a rate of < 300 events per second on a Helios mass cytometer

(Fluidigm) equipped with a modified wide-bore injector (Fluidigm). The FCS files of the acquired events were normalized and concatenated with Fluidigm acquisition software and deconvoluted with a MATLAB-based debarcoding application. The resulting files were analyzed with OMIQ software.

Deep immunophenotyping by mass cytometry (CyTOF)

CyTOF was performed on whole blood from adult and pediatric healthy controls, P4, P5, P6 and an IL-12Rβ1-deficient patient (Table S3), with the Maxpar Direct Immune Profiling Assay (Fluidigm), according to the manufacturer's instructions. Cells were frozen at -80°C after the overnight dead-cell staining step, and acquisition was performed on a Helios machine (Fluidigm). All the samples were processed within 48 hours of sampling. The data were analyzed with OMIQ software.

Deep immunophenotyping by spectral flow cytometry

Deep immunophenotyping by spectral flow cytometry was performed as previously described (*87*). Briefly, freshly thawed PBMCs from adult and pediatric healthy controls, P2, P3, P4, three IL-12Rβ1-deficient and four STAT1-GOF patients (Table S3) were stained with LIVE/DEAD Fixable Blue (Invitrogen, Cat: L23105, 1:800 in PBS) and for cell-surface antigens (*87*). The cells were then washed, fixed in 1% paraformaldehyde (PFA) in PBS, washed again, and acquired with an Aurora cytometer (Cytek). Subsets were manually gated with FlowJo.

Single-cell RNA sequencing of primary leukocytes at steady state

Cryopreserved PBMCs from P3, P4 and P6 sampled after the complete remission of BCG infection or CMC were analyzed in three different batches of experiments, by single-cell

RNA sequencing (scRNASeq). Two IL-12Rβ1-deficient patients, two IL-12Rβ2-deficient patients and one STAT1-GOF patient were simultaneously analyzed in two of the three batches (Table S3). Briefly, thawed cells were washed with medium and filtered with a 70 μm MACS SmartStrainer (Miltenyi Biotec, Cat: 130-098-462) to remove large debris. The cells were then washed three times with 0.5% FBS in PBS and filtered again with a Falcon 40 μm-mesh Cell Strainer (Corning, Cat: 352340) before capture with the 10X Genomics Chromium chip. Libraries were prepared with Chromium Single Cell 3' Reagent Kit (v3 Chemistry) and sequenced with an Illumina NovaSeq 6000 sequencer. Sequences were preprocessed with Cell Ranger.

The data generated during this study were analyzed in an integrative manner, with historical controls from the laboratory (nine adult and four pediatric controls in six batches of experiments) and publicly available control PBMC datasets downloaded from the 10X Genomics web portal (https://support.10xgenomics.com/single-cell-gene-expression/datasets). Data were first manually inspected to check the percentage of mitochondrial genes, the number of mRNA molecules, and the number of detected genes per cell. Curated datasets were integrated with Harmony (*88*) to remove batch effects. Two sequential rounds of graph-based clustering were performed. The second-round clustering focused on memory and effector T cells and NK cells to achieve T-cell subset identification with sufficient resolution. Clusters were manually identified with the SingleR pipeline (*78*), guided by the normalized RNA sequencing dataset generated by Monaco *et al.* (*79*), and the manual inspection of representative marker genes. The TotalSeq datasets derived from 10X also provided information about the identity of each cluster. Gene expression was quantified at single-cell level with Seurat (*89*). Pseudobulk differential expression analysis was conducted with DESeq2 (*90*), excluding all public datasets. Principal component analysis was performed by VST transformation implemented in DESeq2, with batch effects removed with the

removeBatchEffect function implemented in limma (*91*). Gene set enrichment analysis (GSEA) was conducted with the fgsea package, by projecting the fold-change ranking onto various MSigDB gene sets (http://www.gsea-msigdb.org/gsea/msigdb/genesets.jsp). Intercellular communication analysis was performed with CellChat (*92*). All analyses were performed in R v4 (http://www.R-project.org/) (*93*).

Single-cell RNA sequencing on PBMCs after IL-23 stimulation

Cryopreserved PBMCs (0.6 x 10^6 cells per condition) from healthy controls (including P4's sister) P4, P6, an IL-12Rβ1-deficient patient and a STAT1-GOF patient were dispensed into a U-bottom 96-well plate and were either left unstimulated or were stimulated with recombinant human IL-23 (100 ng/mL, 1290-IL, R&D Systems) for six hours (Table S3). The stimulated cells were washed three times, filtered with a Falcon 40 μm-mesh cell strainer, and captured with a 10X Chromium chip. Libraries were prepared and sequenced, and the data were preprocessed as described above. Variations due to experimental batch, genotype, and stimulation were integrated with Harmony (*88*) and two sequential clustering analyses were performed, as described in the steady-state scRNASeq section. Gene expression was quantified at single-cell level with Seurat (*89*). Pseudobulk differential expression analysis was conducted with DESeq2 (*90*). Gene set enrichment analysis (GSEA) was conducted with the fgsea package, by projecting the fold-change ranking onto various MSigDB gene sets (http://www.gsea-msigdb.org/gsea/msigdb/genesets.jsp). All analyses were performed in R v4 (http://www.R-project.org/) (*93*).

PBMC stimulation with BCG

Freshly thawed PBMCs from healthy controls, P3, P4, P6, an IL-12Rβ1-deficient patient and a STAT1-GOF patient were dispensed into a U-bottomed 96-well plate at a density

of 2 × 10^5 cells per well, in 200 μL lymphocyte medium per well (Table S3). Cells were incubated in the presence or absence of live BCG, at a multiplicity of infection of 1, with or without recombinant human IL-12 (500 pg/mL, R&D) or recombinant human IL-23 (100 ng/mL, 1290-IL R&D Systems). After 40 hours of stimulation, GolgiPlug (BD Biosciences, 555029; 1:1,000 dilution) was added to each well to inhibit cytokine secretion. After another eight hours of incubation, the cells were collected by centrifugation for flow cytometry analysis. In brief, cells were stained with the Zombie NIR Fixable Viability Kit (BioLegend; 1:2,000 dilution) at room temperature for 15 minutes, and then stained on ice for 30 minutes with a surface-staining panel containing FcR blocking reagent (Miltenyi Biotec; 1:50 dilution), anti-CD3-Alexa Fluor 532 (eBioscience, Clone: UCHT1, 58-0038-42; 1:50 dilution), anti-γδTCR-FITC (eBioscience, Clone:B1.1, 11-9959-41; 1:50 dilution), anti-Vδ2-APC/Fire 750 (BioLegend, Clone:B6, 331419; 1:100 dilution), anti-CD56-BV605 (BioLegend, Clone: 5.1H11, 362537; 1:100 dilution), anti-CD4-BV750 (BD Biosciences, Clone: SK3, 566356; 1:800 dilution), anti-CD8a-Pacific Blue (BioLegend, Clone: SK1, 344717; 1:100 dilution), anti-Vα7.2 TCR-APC (BioLegend, Clone: 3C10, 351708; 1:100 dilution), anti-Vα24-Jα18-PE/Cy7 BioLegend, Clone: 6B11, 342912; 1:100 dilution), anti-CD20-BV785 (BioLegend, Clone: 2H7, 302356; 1:200 dilution) and anti-PD- 1-PE (eBioscience, Clone: MIH4, 12-9969-42; 1:100 dilution) antibodies. Cells were fixed by incubation with 2% paraformaldehyde in PBS on ice for 15 minutes. Cells were then permeabilized/stained by incubation overnight at −20°C in the permeabilization buffer from the Nuclear Transcription Factor Buffer Set (BioLegend), with an intracellular cytokine panel containing FcR blocking reagent (Miltenyi Biotec; 1:50 dilution), anti-IFN-γ-BV711 (BioLegend, Clone: 4 S.B3, 502540; 1:50 dilution), anti-TNF-BV510 (BioLegend, Clone: MAb11, 502950; 1:50 dilution) and anti-IL-10-PE/Dazzle594 (BioLegend, Clone: JES3-19F1, 506812; 1:50 dilution) antibodies. As a positive control, cells in a separate well were stimulated by incubation with phorbol-12-myristate-13-

acetate (PMA, Sigma; 25 ng mL^{-1}) and ionomycin (Sigma; 500 nM) for one hour without GolgiPlug followed by a further 7 hours with GolgiPlug (for intracellular cytokine staining). Cells were acquired with an Aurora cytometer (Cytek). Data were manually gated with FlowJo.

Ex vivo naïve and memory $CD4^+$ T-cell stimulation

Freshly thawed PBMCs were labeled with anti-CD4-APC-Cy7 (BD Pharmingen, Cat.: 557871, Clone: RPA-T4, 1/50), anti-CD45RA-PerCPCy5.5 (Thermo Fisher Scientific, Cat.: 45045842, Clone: HI100, 1/100), anti-CCR7-FITC (R&D Systems, Cat.: FAB197F100, Clone: 150503, 1/10), anti-CD25-PE-Cy7 (BD Pharmingen, Cat.: 557741, Clone: M-A251, 1/80), and anti-CD127-BV650 (Biolegend, Cat.: 351326, Clone: A019D5, 1/50) antibodies. Following the exclusion of T_{reg} ($CD127^{lo}CD25^{hi}$ $CD4^+$) cells, we isolated naïve (defined as $CD45RA^+$ $CCR7^+$ $CD4^+$) or effector/memory (defined as $CD45RA^-$ $CCR7^-$ $CD4^+$) T cells (>98% purity) with a FACS Aria cell sorter (BD Biosciences). Purified naïve or effector/memory $CD4^+$ T cells were cultured with T-cell activation and expansion (TAE) beads (anti-CD2/CD3/CD28, Miltenyi Biotec) alone (T_H0), or under T_H1 (IL-12 [50 ng/mL; R&D Systems]), T_H2 (IL-4 [1 U/mL; eBioscience]), T_H17 (TGFβ [2.5 ng/mL; R&D Systems], IL-1β [50 ng/mL; Peprotech], IL-6 [50 ng/mL; PeproTech], IL-21 [50 ng/mL; PeproTech], IL-23 [50 ng/mL; eBioscience]) or T_H9 (IL-9 [100 U/mL; eBioscience], TGFβ [2.5 ng/mL; R&D Systems]) polarizing conditions. After five days, culture supernatants were collected for evaluation of the secretion of IL-2, IL-4, IL-9, IL-10, IL-17A, IL-17F, and IFN-γ in a cytometric bead array (BD Biosciences). Once the supernatants had been collected, the cells were stimulated with PMA (100 ng/mL, Sigma-Aldrich)-ionomycin (750 ng/mL, Sigma-Aldrich) for six hours, with brefeldin A (10 μg/mL, Sigma-Aldrich) added after two hours of stimulation. We assessed intracellular cytokine production by staining cells with anti-IFN-γ-BV605 (BD Horizon, Cat.: 562974, Clone: B27, 1/40), anti-IL-9-PerCPCy5.5 (BD Pharmingen, Cat.: 561461, Clone:

MH9A3, 1/20), anti-IL-4-PE-Cy7 (Thermo Fisher Scientific, Cat.: 25704982, Clone 8D4-8, 1/100), anti-IL-17A-APC-Cy7 (BioLegend, Cat.: 512320, Clone: BL168, 1/50), anti-IL-17F-BV786 (BD Horizon, Cat.: 564265, Clone: O33-782, 1/30), and anti-IL-2-BV711 (BD Horizon, Cat.: 563946, Clone: 5344.111, 1/50) antibodies. Cells were acquired with a BD LSRFortessa™ Cell Analyzer (BD Biosciences). Data were manually gated with FlowJo software (Tree Star), with a gating strategy described elsewhere (*95*, *96*).

***Ex vivo* evaluation of BCG-reactive memory CD4$^+$ T cells**

Fresh PBMCs were dispensed into a U-bottomed 96-well plate at a density of 1 x 10^6 cells per well, in 200 uL of RPMI-1640 medium supplemented with 10% human serum (Sigma-Aldrich). Cells were left unstimulated or were incubated with tuberculin PPD (5 µg/mL, Statens Serum Institut) or ImmunoCult™ Human CD3/CD28/CD2 T-cell activator (25 µL/mL). After six hours of incubation, with brefeldin A (GolgiPlug, BD Biosciences, 1/1000 final concentration) added for the last four hours, the cells were stained with anti-CCR7-APC (Miltenyi, Cat.: 130 120 460, Clone: REA108, 1/50), anti-CD8-BV650 (BD Biosciences, Cat.: 563821, Clone: RPA-T8, 1/50), anti-CD4-BV711 (BD Biosciences, Cat.: 563028, Clone: SK3, 1/50), anti-CD45-RA-PE-CF594 (BD Biosciences, Cat.: 562298, Clone: HI100, 1/100) antibodies, Aqua Dead cell marker (Thermo Fisher Scientific) and FcR blocking reagent (Miltenyi Biotec; 1:50 dilution) for 30 min at 4°C. The cells were then incubated for 15 min at room temperature in Fixation/Permeabilization buffer (Invitrogen), and stained by overnight incubation with anti-IFN-γ-AF700 (BD Biosciences, Cat.: 557995, Clone: B27, 1/100), anti-CD154-BV421 (BioLegend, Cat.: 310824, Clone: 24-31, 1/50), anti-CD3-BV786 (BD Biosciences, Cat.: 563800, Clone: SK7, 1/50), anti-CD69-PE-Cy7 (BioLegend, Cat.: 310912, Clone FN50, 1/25) antibodies and FcR blocking reagent (Miltenyi Biotec; 1:50 dilution) at

4°C. Cells were acquired with a Fortessa X20 (BD Biosciences). Data were manually gated with FlowJo.

T-cell libraries

Cryopreserved PBMCs from patients and healthy controls were stained with the following staining panel: CD4-PE-Dazzle594 (BioLegend, Cat.: 300547, Clone RPA-T4, 1:800), CD45RA-Qdot655 (Invitrogen, Cat.: Q10069, Clone: MEM-56, 1:800), CCR7-BV421 (BioLegend, Cat.: 353208, Clone: G043H7, 1:80), CD95-APC-Fire750 (BioLegend, Cat.: 305637, Clone: DX2, 1:200), CD19-AF700 (BioLegend, Cat.: 302225, Clone: HIB19, 1:200), CCR6-BV605 (BioLegend, Cat.: 353420, Clone: G034E3, 1:80), CCR4-PE-Cy7 (BD Biosciences, Cat.: 557864, Clone 1G1, 1:80), CXCR3-AF647 (BioLegend, Cat.: 353711, Clone: G025H7, 1:80), CD14-PE-Cy5 (Beckman Coulter, Cat.: A0776, Clone: RMO52, 1:30), CD56-PE-Cy5 (Beckman Coulter, Cat.: A07789, Clone N901, 1:30), CD8-FITC (Beckman Coulter, Cat.: A07758, Clone: B9.11, 1:30), CD25-PE (BD Biosciences, Cat.: 555432, Clone: M-A251, 1:20). Viability was assessed with SytoxGreen (Cat.: S34860, Thermo Fisher Scientific) according to the manufacturer's instructions. Naïve $CD4^+$ T cells (defined as $CD45RA^+$ $CCR7^+$ $CD4^+$ $CD95^-$) and memory $CD4^+$ T cells (excluding $CD25^{high}$ and $CD45RA^+$ $CCR7^+$ cells) were isolated (>98% purity) with a FACSAria Fusion cell sorter (BD Biosciences). Memory T-cell library screening was performed as previously described (*97*). Briefly, memory T cells were used to seed 96-well culture plates at a density of 500 cells/well in complete RPMI medium (Cat.: 31870025, Gibco) supplemented with GlutaMAX (2 mM, Cat.: 35050087, Invitrogen), non-essential amino acids (1% [vol/vol], Cat.: 11140035, Invitrogen), sodium pyruvate (1 mM, Cat.: 11360039, Invitrogen), penicillin (50 U/mL) and streptomycin (50 μg/mL) (Cat.: 15070063, Invitrogen) and 5% human serum (Swiss Red Cross). Cells were stimulated polyclonally with PHA (1 μg/mL, Cat.: R30852801, Thermo

Fisher Scientific) in the presence of irradiated (45 Gy) allogeneic PBMCs as feeder cells (1×10^5 per well) and IL-2 (500 IU/mL), and T-cell lines were expanded by incubation in the presence of IL-2. The T-cell lines were screened 21-28 days after initial stimulation, by culturing thoroughly washed T cells (2.5×10^5/well) with autologous irradiated B cells (2.5×10^4/well), with or without a three-hour pulse with various antigens, including an *M. tuberculosis* peptide pool (0.5 μg/mL/peptide, comprising 207 peptides), a BCG peptide pool (0.5 μg/mL/peptide, comprising 211 peptides), a CMV peptide pool (0.5 μg/mL/peptide, comprising 76 peptides), an EBV peptide pool (0.5 μg/mL/peptide, comprising 46 peptides), an influenza virus peptide pool (0.5 μg/mL/peptide, comprising 112 peptides spanning hemagglutinin (A&A Labs) and 91 peptides spanning neuraminidase) (all kindly provided by Alessandro Sette, LJI), and a *C. albicans* peptide pool (0.5 μg/mL/peptide, comprising 252 peptides) (Pepscan). Proliferation was assessed on day 4, after incubation for 16 hours with 2 μCi/mL [3H]-thymidine (Cat.: NET027005MC, Perkin Elmer). Cytokine concentrations in culture supernatants were determined after 48 hours of stimulation, in Luminex multiplex cytokine assays (Thermo Fisher Scientific). Precursor frequencies were calculated from the number of negative wells, assuming a Poisson distribution, and are expressed per million cells. The responding helper T-cell subset was identified by sorting antigen-reactive T-cell lines into CCR6$^+$ (comprising T_H17 and T_H1^* cells) and CCR6$^-$ (comprising T_H1 and T_H2 cells) compartments with a FACSAria Fusion cell sorter (BD Biosciences) and the following antibody panel: CCR6-BV605 (BioLegend, Cat.: 353420, Clone: G034E3, 1:80), CCR4-PE-Cy7 (BD Biosciences, Cat.: 557864, Clone: 1G1, 1:80), CXCR3-AF647 (BioLegend, Cat.: 353711, Clone: G025H7, 1:80). CCR6$^+$ and CCR6$^-$ lines were screened with peptides as described, with autologous EBV-B cells (2.5×10^4/well) as antigen-presenting cells and 1.5×10^5 T cells/well.

Stimulation of fresh PBMCs with HKCA

Fresh PBMCs from healthy controls, P5, P6 and one IL-12Rβ1-deficient patient were dispensed into a U-bottomed 96-well plate at a density of 1×10^5 cells per well, in 100 μL of RPMI-1640 medium supplemented with 10% human serum (Sigma-Aldrich) and IL-2 (0.05 ng/mL, Novartis) (Table S3). Cells were incubated with or without IL-1β (2.5 ng/mL, R&D) and/or IL-23 (100 ng/mL, R&D), in the presence or absence of HKCA (1×10^5 cells per well, InvivoGen). After five days of incubation, the supernatant was collected. The concentrations of IL-17A, IL-17F and IL-22 in the supernatant were determined with the LEGENDplex™ T Helper Cytokine Panel Version 2 kit (BioLegend, 740510).

Stimulation of freshly thawed PBMCs with HKCA

Freshly thawed PBMCs from healthy controls, P3, P4, P6 and two IL-12Rβ1-deficient patients were dispensed into a U-bottomed 96-well plate at a density of 1×10^5 cells per well, in 100 μL RPMI-1640 medium supplemented with 10% human serum (Sigma-Aldrich) and IL-2 (10 ng/mL, Novartis) (Table S3). Cells were incubated in the presence or absence of HKCA (1×10^5 cells per well, InvivoGen). After seven days of incubation, the supernatant was collected. The concentrations of IL-17A and IL-17F in the supernatant were determined with the LEGENDplex™ T Helper Cytokine Panel Version 2 kit (BioLegend, 740510).

Statistical analysis

The statistical analyses for Figures 4E, Figure 6A and B, and Figure S6A and B were performed with GraphPad Prism 8.4.3. Mann-Whitney nonparametric tests were used for analysis. A P value <0.05 was considered statistically significant.

Figure S1

A

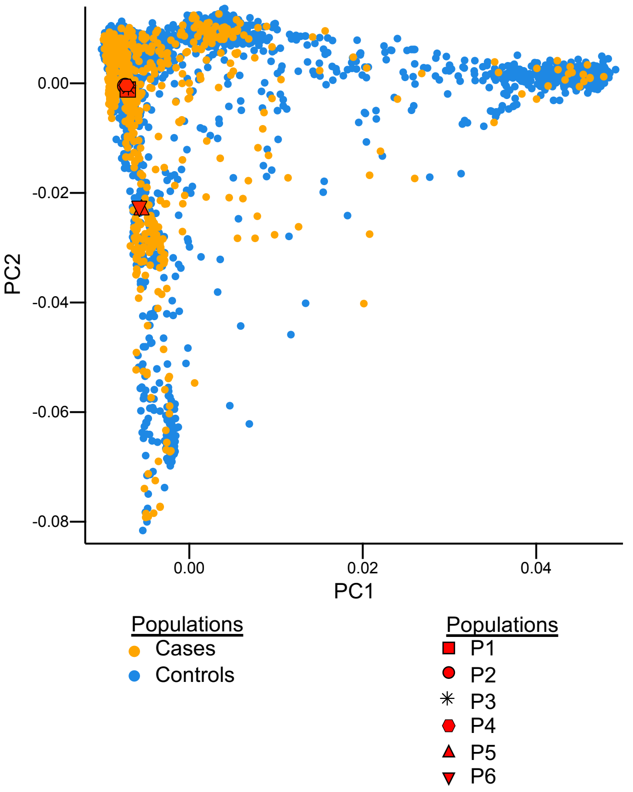

C

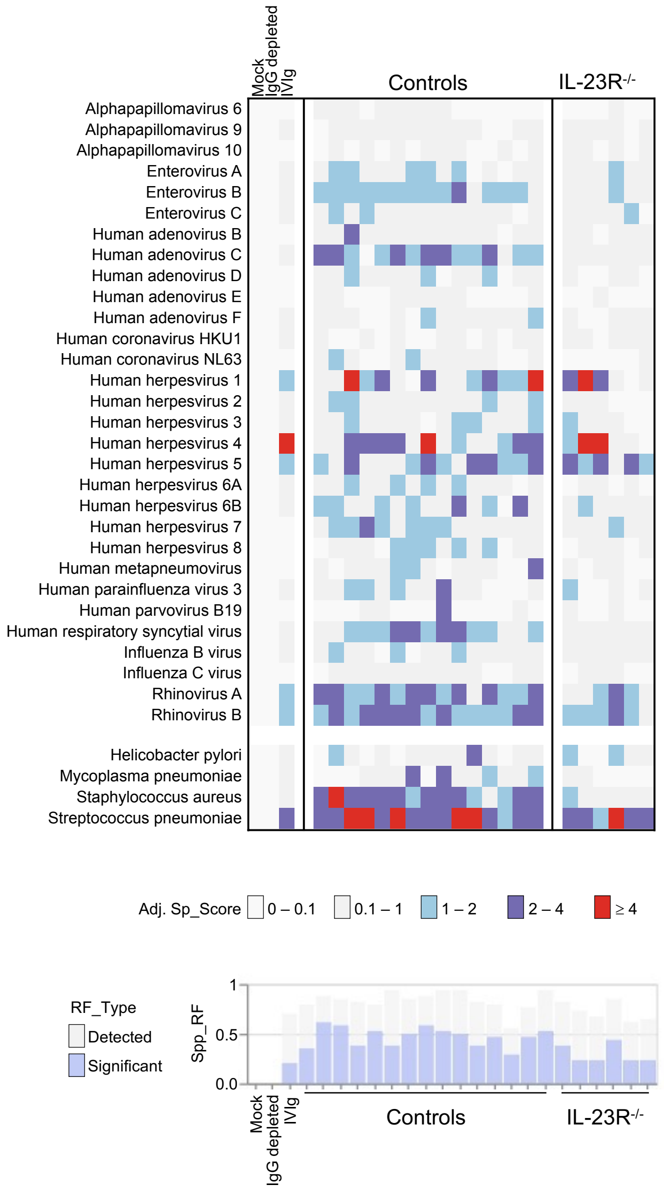

B

	P1	P2	P3	P4	P5	P6
Homozygosity rate	2.55%	1.74%	2.25%	2.61%	3.50%	3.06%
Total number of variants DP ≥ 5 – MQ ≥ 30	12,030	7,783	16,828	10,231	11,477	11,583
Number of variants MAF < 0.01 in gnomAD	6,557	4,492	1,004	5,770	7,139	7,107
Homozygous variants coding, essential splice-site, splice-site CADD > MSC	27	21	27	76	51	51
Not present in the homozygous / hemizygous state in GnomAD v2.1.1	11	10	15	28	20	17
Shared gene	*IL23R*					
Variant	p.C115Y		c.367+ 1G>A	c.1149 -1G>A	p.E269*	

D

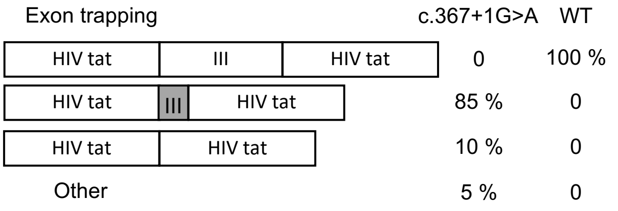

E

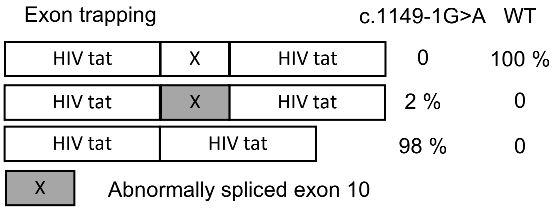

Figure S1

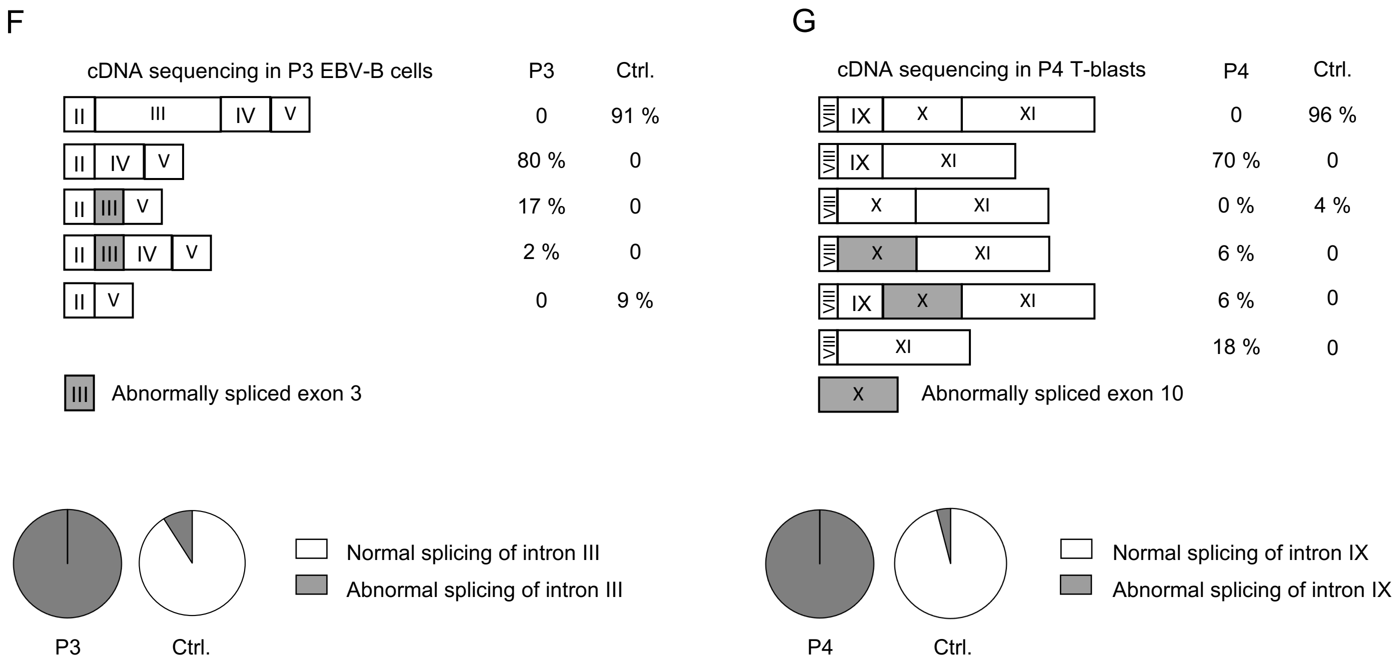
F
cDNA sequencing in P3 EBV-B cells
P3
Ctrl.
II III IV V
0
91 %
II IV V
80 %
0
II III V
17 %
0
II III IV V
2 %
0
II V
0
9 %
III Abnormally spliced exon 3
Normal splicing of intron III
Abnormal splicing of intron III
P3
Ctrl.
G
cDNA sequencing in P4 T-blasts
P4
Ctrl.
VIII IX X XI
0
96 %
VIII IX XI
70 %
0
VIII X XI
0 %
4 %
VIII X XI
6 %
0
VIII IX X XI
6 %
0
VIII XI
18 %
0
X Abnormally spliced exon 10
Normal splicing of intron IX
Abnormal splicing of intron IX
P4
Ctrl.

Supplementary Figure 1: Private homozygous *IL23R* variants in four Iranian kindreds

(**A**) Principal component analysis (PCA) was performed as explained in the Materials and Methods, with data for 802 MSMD patients with no identified genetic etiology (cases), and 3,410 patients of diverse ethnic origins from our in-house database presenting with various infectious diseases, excluding mycobacterial or fungal diseases (controls). The six patients with homozygous experimentally proven loss-of-function (eLOF) or predicted LOF *IL23R* variants are shown. P1 to P4 are located within clusters of individuals from our in-house database of European origin, whereas P5 and P6 are located within a cluster of individuals of Iranian origin. **(B)** Homozygosity rate estimated from WES data and with the variant filtering strategy, for patients P1 to P6. (**C**) VirScan assay showing the presence of antibodies against several viruses and bacteria in the serum samples from 15 healthy individuals, and the six IL-23R-deficient patients. Adj Sp_score: adjusted species score; spp_RF, significant species response frequency. (**D-E**) Schematic representation of *IL23R* transcripts and their relative frequencies by exon trapping in COS-7 cells. (**F-G**) Schematic representation of *IL23R* transcripts and their relative frequencies after cDNA sequencing in EBV-B cells from P3 and a control (**F**), and in T-cell blasts (T-blasts) from P4 and a control **(G)**.

Figure S2

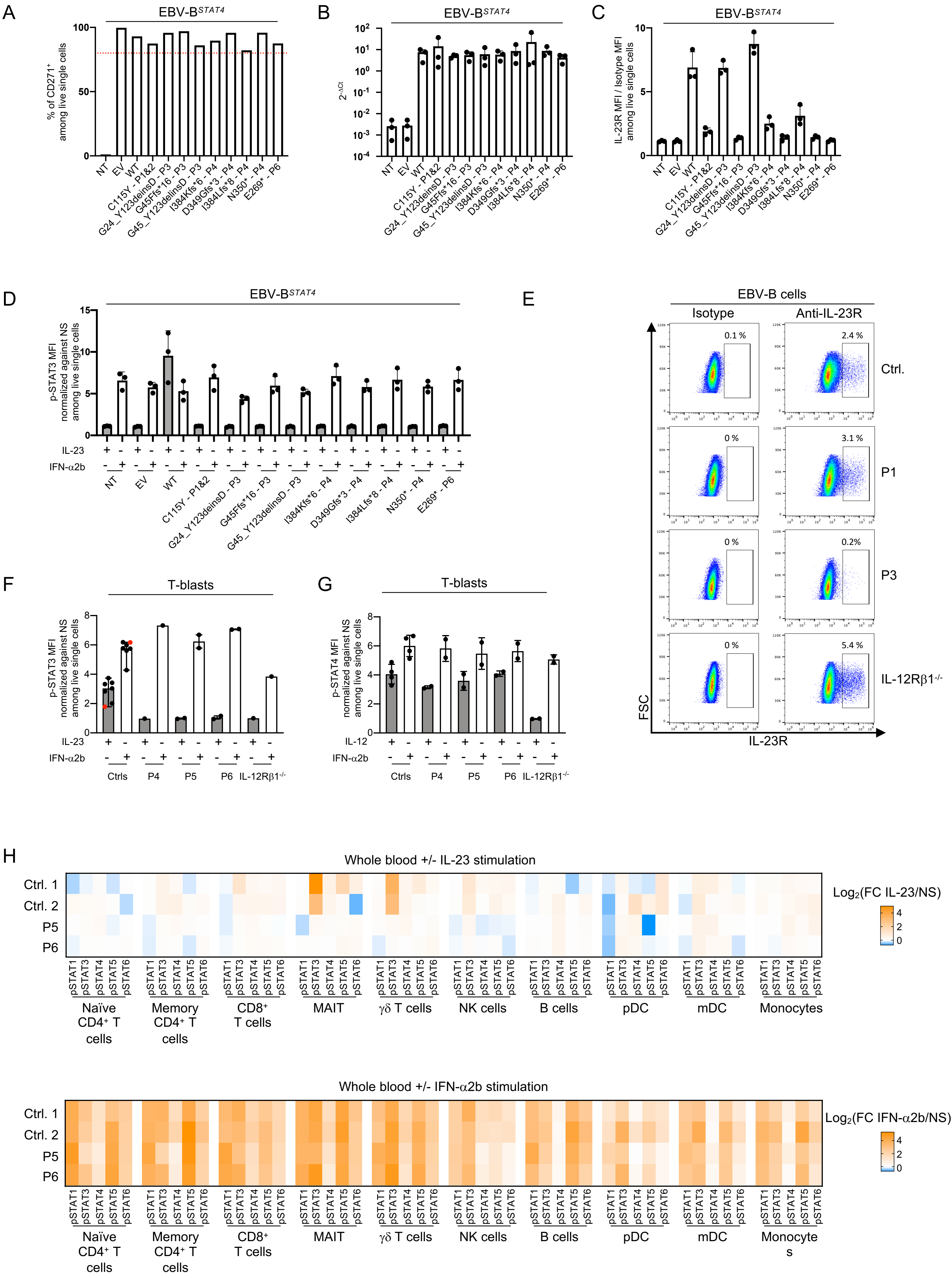

A
EBV-B^{STAT4}
% of CD271+ among live single cells
NT
EV
WT
C115Y - P1&2
G24_Y123deinsD - P3
G45Ffs*16 - P3
G45_Y123delinsD - P3
I384Kfs*6 - P4
D349Gfs*3 - P4
I384Lfs*8 - P4
N350* - P4
E269* - P6
B
$2^{-\Delta Ct}$
C
IL-23R MFI / Isotype MFI among live single cells
D
p-STAT3 MFI normalized against NS among live single cells
IL-23
IFN-α2b
E
EBV-B cells
Isotype
Anti-IL-23R
Ctrl.
P1
P3
IL-12Rβ1-/-
FSC
IL-23R
F
T-blasts
Ctrls
P4
P5
P6
G
p-STAT4 MFI normalized against NS among live single cells
IL-12
H
Whole blood +/- IL-23 stimulation
Log2(FC IL-23/NS)
Ctrl. 1
Ctrl. 2
pSTAT1
pSTAT3
pSTAT4
pSTAT5
pSTAT6
Naïve CD4+ T cells
Memory CD4+ T cells
CD8+ T cells
MAIT
γδ T cells
NK cells
B cells
pDC
mDC
Monocytes
Whole blood +/- IFN-α2b stimulation
Log2(FC IFN-α2b/NS)

Supplementary Figure 2: Loss-of-function *IL23R* alleles and AR complete *IL23R* deficiency

(**A-D**) EBV-B^{STAT4} cells were either left non-transduced (NT) or were transduced with lentiviruses generated with an empty vector (EV) or vectors containing the WT or the mutated *IL23R* cDNA. (**A**) Cell-surface NGFR (CD271) expression was assessed by flow cytometry as a surrogate for cell transduction. **(B)** *IL23R* mRNA levels were determined by RT-qPCR, and normalized against *GUSB* mRNA levels by the $2^{-\text{delta CT}}$ method. **(C)** Cell-surface IL-23R expression was assessed by flow cytometry. The data in panel **C** are presented as the ratio of the mean fluorescence intensity (MFI) of the IL-23R-specific antibody to the MFI of the isotype control antibody. Non-transduced (NT) or transduced EBV-B^{STAT4} cells were left unstimulated (NS), or were stimulated with IL-23 or IFN-α2b as a positive control. STAT3 phosphorylation was assessed by flow cytometry (**D**). The data in panel **D** are presented as the ratio of the MFI of phosphorylated STAT3 after IL-23 or IFN-α2b stimulation to the MFI of phosphorylated STAT3 in unstimulated cells. The results of three independent experiments are depicted in **A-D**. (**E**) Cell-surface IL-23R expression was assessed by flow cytometry in EBV-B cells from a healthy control (Ctrl.), P1, P3 and an IL-12Rβ1-deficient patient. EBV-B cells were stained with an isotype control antibody (Isotype) or with an antibody against IL-23R (Anti-IL-23R). (**F**) T-blasts from seven healthy controls (Ctrls), including P4's sister (red dots), P4, P5, P6 and an IL-12Rβ1-deficient patient were left unstimulated, or were stimulated with IL-23 (+) or IFN-α2b (+) as a positive control. STAT3 phosphorylation was evaluated by flow cytometry. (**G**) T-blasts from four healthy controls (Ctrls), P4, P5, P6, and an IL-12Rβ1-deficient patient were left unstimulated, or were stimulated with IL-12 (+) or IFN-α2b (+) as a positive control. STAT4 phosphorylation was evaluated by flow cytometry. In **(D)**, **(F),** and **(G)**, data are presented as the ratio of the MFI of p-STAT3 after the indicated stimulation to the MFI of pSTAT3 without stimulation. (**H**) Whole blood from two healthy controls (Ctrl. 1

and 2), P5, and P6 was left unstimulated, or was stimulated with IL-23 (top panel) or IFN-α2b (bottom panel) as a positive control. The phosphorylation of STAT1, STAT3, STAT4, STAT5, and STAT6 in the various cell subsets was assessed by pSTAT CyTOF. The $\log_2$ fold changes of pSTAT signal intensity between the cells stimulated with IL-23 or IFN-α2b and those left unstimulated (NS) [Log_2(FC IL-23/NS) and Log_2(FC IFN-α2/NS), respectively] in the various cell subsets are depicted in heatmaps.

Figure S3

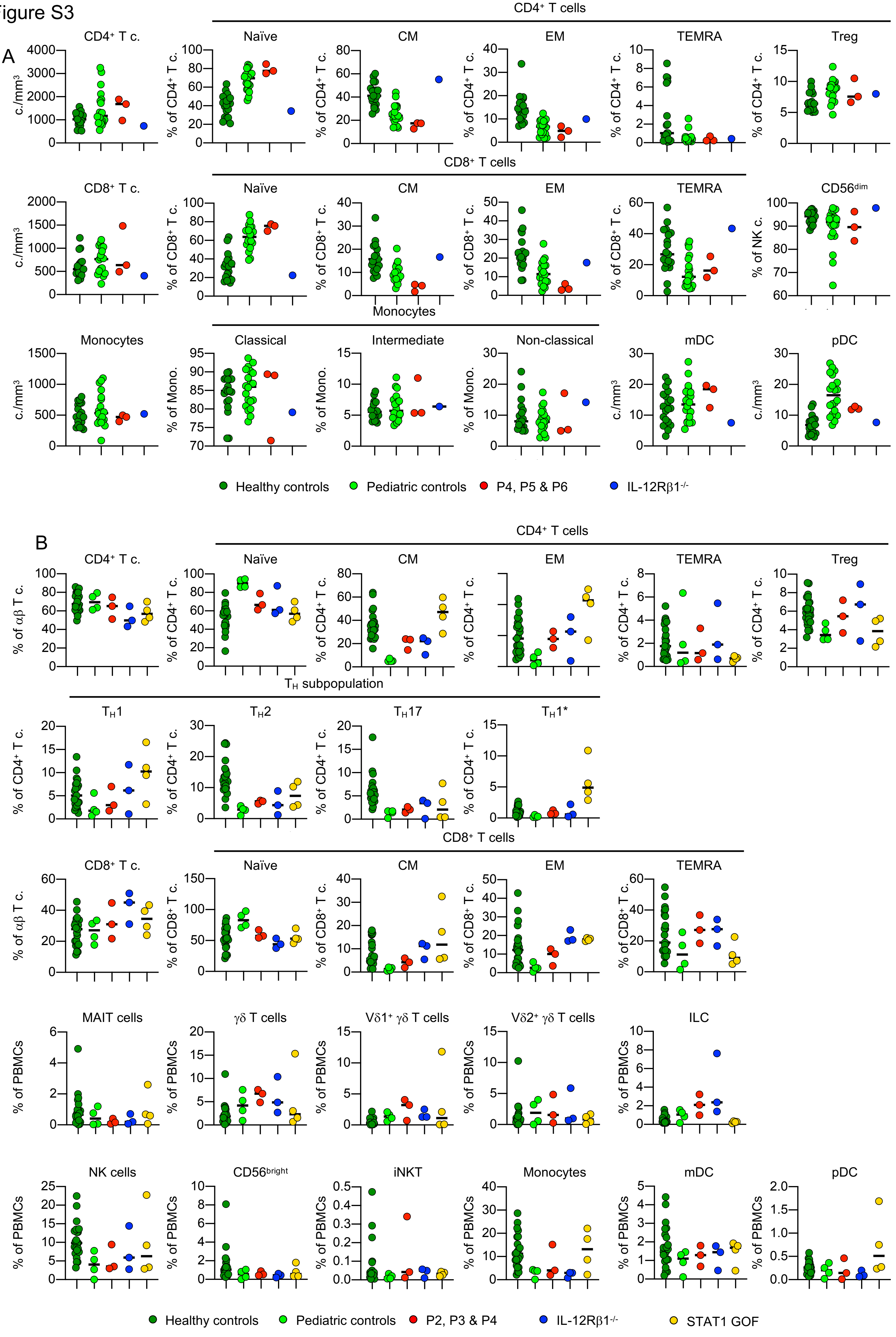
A
CD4+ T cells
CD4+ T c.
Naïve
CM
EM
TEMRA
Treg
c./mm3
% of CD4+ T c.
CD8+ T cells
CD8+ T c.
CD56dim
% of CD8+ T c.
% of NK c.
Monocytes
Classical
Intermediate
Non-classical
mDC
pDC
% of Mono.
Healthy controls
Pediatric controls
P4, P5 & P6
IL-12Rβ1-/-
B
% of αβ T c.
TH subpopulation
TH1
TH2
TH17
TH1*
MAIT cells
γδ T cells
Vδ1+ γδ T cells
Vδ2+ γδ T cells
ILC
% of PBMCs
NK cells
CD56bright
iNKT
P2, P3 & P4
STAT1 GOF

Figure S3

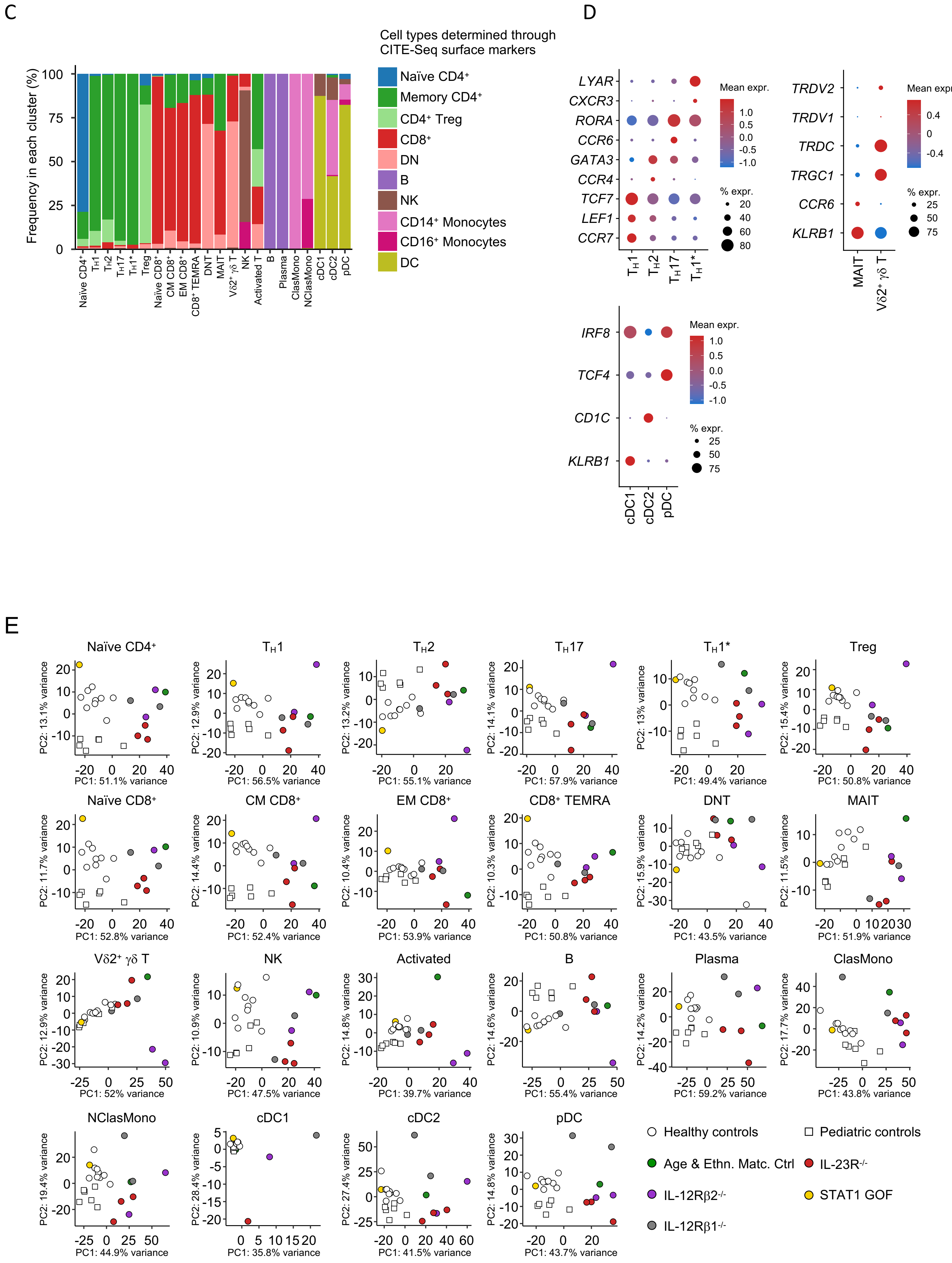
C
Cell types determined through CITE-Seq surface markers
Frequency in each cluster (%)
Naïve CD4+
Memory CD4+
CD4+ Treg
CD8+
DN
B
NK
CD14+ Monocytes
CD16+ Monocytes
DC
D
Mean expr.
% expr.
LYAR
CXCR3
RORA
CCR6
GATA3
CCR4
TCF7
LEF1
CCR7
TRDV2
TRDV1
TRDC
TRGC1
KLRB1
MAIT
Vδ2+ γδ T
IRF8
TCF4
CD1C
cDC1
cDC2
pDC
E
Naïve CD4+
TH1
TH2
TH17
TH1*
Treg
Naïve CD8+
CM CD8+
EM CD8+
CD8+ TEMRA
DNT
MAIT
Vδ2+ γδ T
NK
Activated
B
Plasma
ClasMono
NClasMono
cDC1
cDC2
pDC
Healthy controls
Pediatric controls
Age & Ethn. Matc. Ctrl
IL-23R-/-
IL-12Rβ2-/-
STAT1 GOF
IL-12Rβ1-/-

Supplementary Figure 3: Development of peripheral mononuclear hematopoietic cells in IL-23R-deficient patients

(**A**) Absolute count of $CD4^+$ T cells, frequency of naïve, memory and Treg $CD4^+$ T cells, absolute counts of $CD8^+$ and memory $CD8^+$ T cells, frequency of $CD56^{dim}$ NK cells, absolute counts of monocytes and dendritic cells in 19 healthy adult controls, 20 healthy pediatric controls, IL-23R-deficient patients (P4, P5, P6), and one IL-12Rβ1-deficient patient, as assessed by CyTOF on whole blood. (**B**) Frequency of $CD4^+$, $CD8^+$, NK, innate-like (MAIT, γδ T and ILC) cells, monocytes, and dendritic cells in 23 healthy controls, four pediatric healthy controls, IL-23R-deficient patients (P4, P5, and P6), three IL-12Rβ1-deficient patients and four STAT1 gain-of-function (GOF) patients, as assessed by spectral flow cytometry on cryopreserved PBMCs. (**C**) Frequencies of cell surface marker-determined cell types, based on data from the CITE-seq database, in each cluster. (**D**) Single-cell expression of representative genes in each cluster. (**E**) Single-cell transcriptome analysis. Pseudobulk principal component analysis (PCA) of the transcriptional phenotypes in each leukocyte subset.

Figure S4

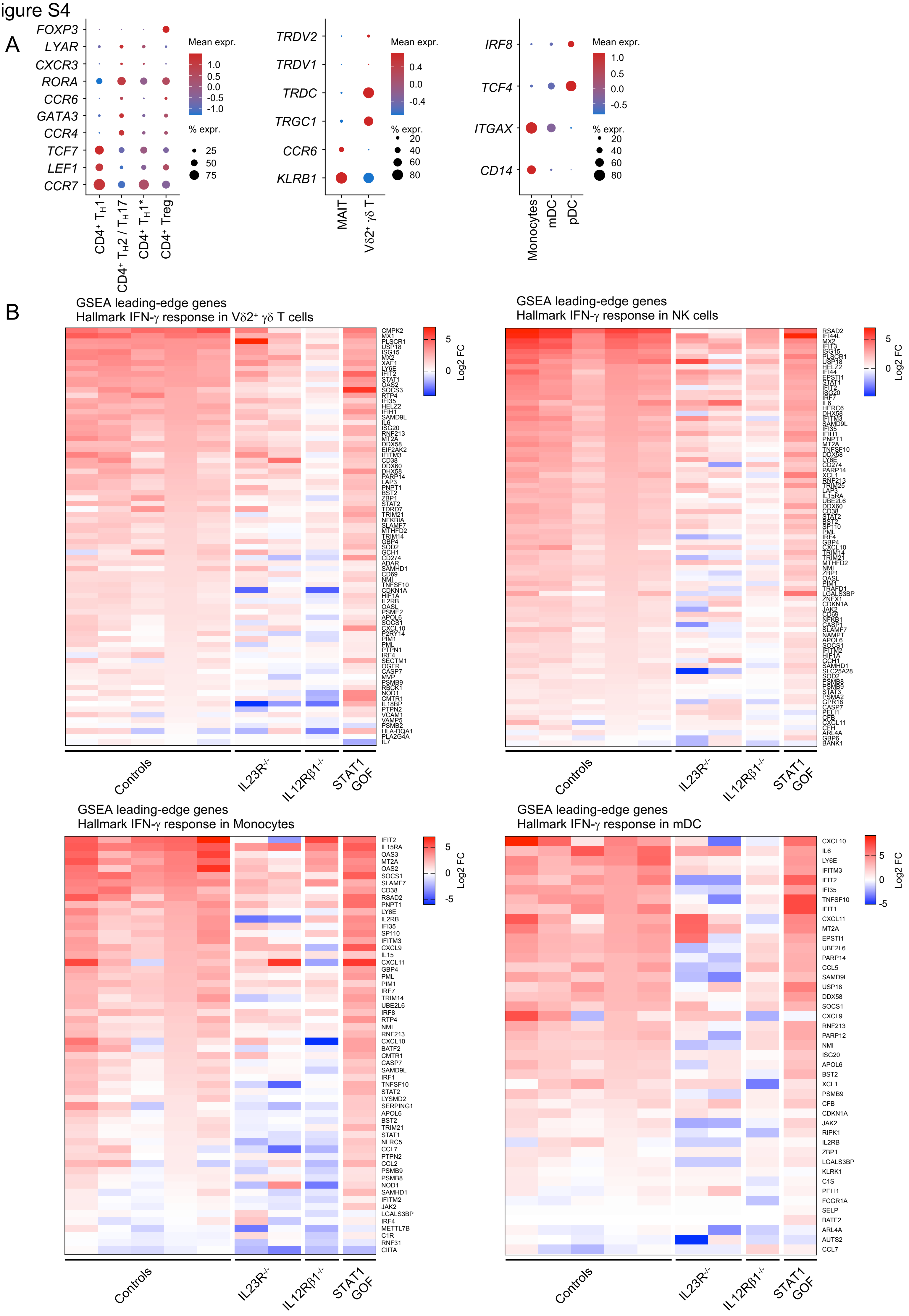
A
FOXP3
LYAR
CXCR3
RORA
CCR6
GATA3
CCR4
TCF7
LEF1
CCR7
Mean expr.
1.0
0.5
0.0
-0.5
-1.0
% expr.
25
50
75
CD4+ TH1
CD4+ TH2 / TH17
CD4+ TH1*
CD4+ Treg
TRDV2
TRDV1
TRDC
TRGC1
CCR6
KLRB1
Mean expr.
0.4
0.0
-0.4
% expr.
20
40
60
80
MAIT
Vδ2+ γδ T
IRF8
TCF4
ITGAX
CD14
Mean expr.
1.0
0.5
0.0
-0.5
% expr.
20
40
60
80
Monocytes
mDC
pDC
B
GSEA leading-edge genes
Hallmark IFN-γ response in Vδ2+ γδ T cells
GSEA leading-edge genes
Hallmark IFN-γ response in NK cells
GSEA leading-edge genes
Hallmark IFN-γ response in Monocytes
IFIT2
IL15RA
OAS3
MT2A
OAS2
SOCS1
SLAMF7
CD38
RSAD2
PNPT1
LY6E
IL2RB
IFI35
SP110
IFITM3
CXCL9
IL15
CXCL11
GBP4
PML
PIM1
IRF7
TRIM14
UBE2L6
IRF8
RTP4
NMI
RNF213
CXCL10
BATF2
CMTR1
CASP7
SAMD9L
IRF1
TNFSF10
STAT2
LYSMD2
SERPING1
APOL6
BST2
TRIM21
STAT1
NLRC5
CCL7
PTPN2
CCL2
PSMB9
PSMB8
NOD1
SAMHD1
IFITM2
JAK2
LGALS3BP
IRF4
METTL7B
C1R
RNF31
CIITA
GSEA leading-edge genes
Hallmark IFN-γ response in mDC
CXCL10
IL6
LY6E
IFITM3
IFIT2
IFI35
TNFSF10
IFIT1
CXCL11
MT2A
EPSTI1
UBE2L6
PARP14
CCL5
SAMD9L
USP18
DDX58
SOCS1
CXCL9
RNF213
PARP12
NMI
ISG20
APOL6
BST2
XCL1
PSMB9
CFB
CDKN1A
JAK2
RIPK1
IL2RB
ZBP1
LGALS3BP
KLRK1
C1S
PELI1
FCGR1A
SELP
BATF2
ARL4A
AUTS2
CCL7
Log2 FC
5
0
-5
Controls
IL23R-/-
IL12Rβ1-/-
STAT1 GOF

Supplementary Figure 4: Impaired *ex vivo* IL-23-mediated production of IFN-γ in cells from patients with IL-23R deficiency

(**A**) Single-cell expression of representative genes in each cluster. (**B**) Pseudobulk differential expression (DE) analysis. Log_2 fold-changes were estimated with DESeq2 for the interaction between the stimulation (non-stim. vs. IL-23) variables. GSEA for the hallmark IFN-γ gene sets was performed on the basis of log_2 fold-change ranking. Differential gene expression in $V\delta2^+$ γδ, NK cells, classical monocytes, and myeloid dendritic cells (mDCs) between non-stimulated and IL-23-stimulated conditions, for healthy control cells, IL-23R- or IL12Rβ1-deficient cells, and STAT1-GOF cells.

Figure S5

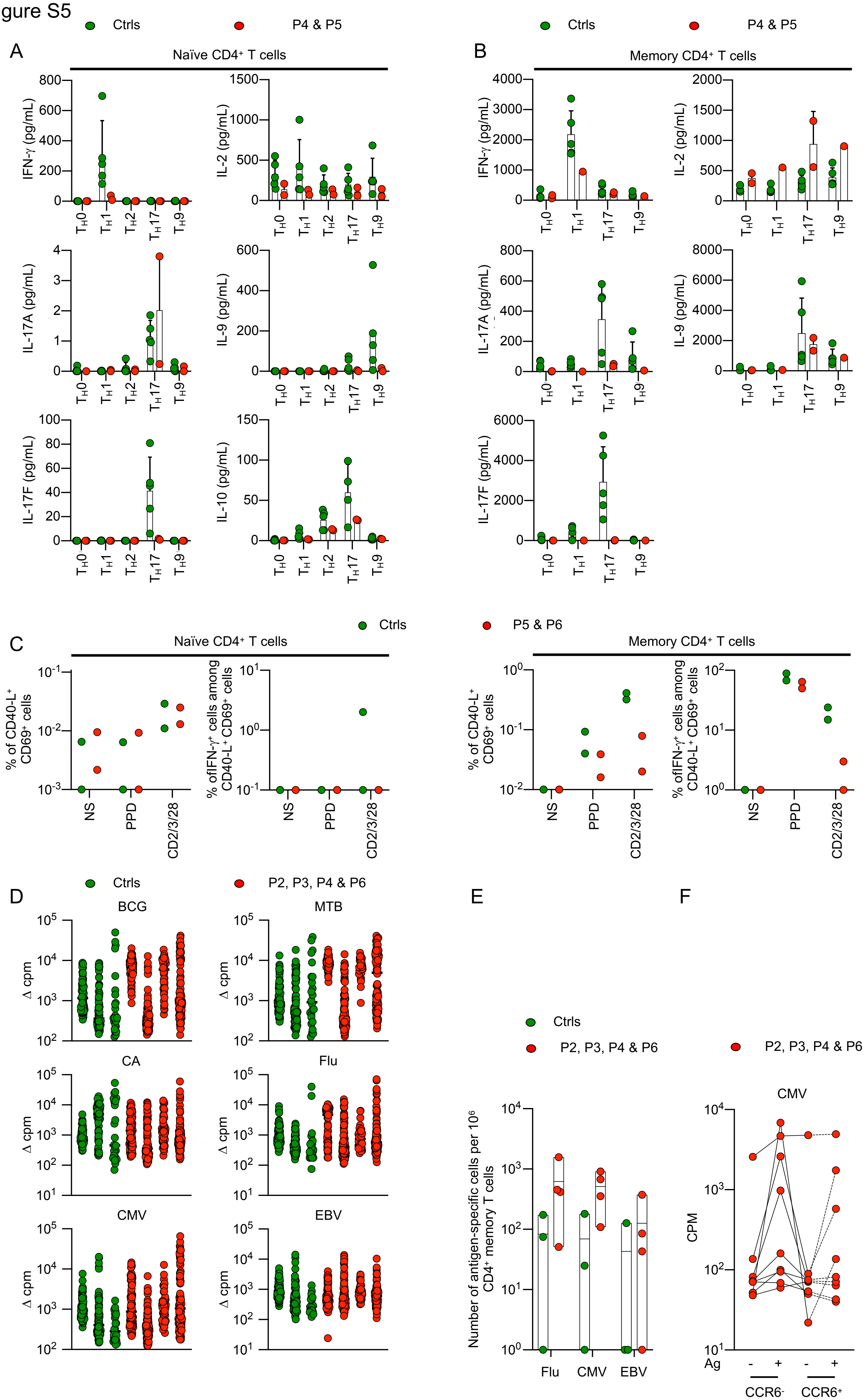
Ctrls
P4 & P5
A
Naïve CD4+ T cells
B
Memory CD4+ T cells
IFN-γ (pg/mL)
IL-2 (pg/mL)
IL-17A (pg/mL)
IL-9 (pg/mL)
IL-17F (pg/mL)
IL-10 (pg/mL)
TH0
TH1
TH2
TH17
TH9
C
P5 & P6
% of CD40-L+ CD69+ cells
% of IFN-γ+ cells among CD40-L+ CD69+ cells
NS
PPD
CD2/3/28
D
P2, P3, P4 & P6
BCG
MTB
CA
Flu
CMV
EBV
Δ cpm
E
F
Number of antigen-specific cells per 10^6 CD4+ memory T cells
Flu
CMV
EBV
CPM
Ag
CCR6-
CCR6+

Supplementary Figure 5: Normal development of BCG- and *C. albicans*-specific memory $CD4^+$ T cells in patients with inherited IL-23R deficiency

(**A-B**) Naïve (**A**) or memory (**B**) $CD4^+$ T cells from healthy controls (Ctrls), P4, and P5 were either left unpolarized (T_H0), or were polarized under T_H1 (T-cell activation/expansion [TAE] beads + IL-12), T_H2 (TAE beads + IL-4), T_H17 (TAE beads + IL-1/IL-6/IL-21/IL-23/TGF-β), or T_H9 (TAE beads + IL-9) conditions. After five days of culture, the levels of IFN-γ, IL-2, IL-17A, IL-9, IL-17F, and IL-10 in the cell culture supernatant were assessed in cytometric bead arrays. (**C**) Fresh PBMCs from two healthy controls (Ctrls) and two IL-23R-deficient patients (P5 and P6) were either left unstimulated (NS), or were stimulated with tuberculin purified protein derivative (PPD), or antibody complexes binding CD2, CD3 and CD28. After six hours of incubation, the proportions of reactive $CD40L^+/CD69^+$ cells, and of IFN-γ^+ reactive cells, among naïve and memory $CD4^+$ T cells, were evaluated by flow cytometry. (**D-F**) Multiple memory $CD4^+$ T-cell lines were generated by the polyclonal stimulation (PHA, IL-2 and irradiated allogeneic PBMCs) of sorted memory $CD4^+$ T cells from three healthy controls, P2, P3, P4, and P6. Lines were screened for reactivity with peptide pools covering antigens from BCG, *Mycobacterium tuberculosis* (MTB), *C. albicans* (CA), influenza virus, CMV and EBV. (**D**) The proliferation of memory $CD4^+$ T-cell lines after stimulation with autologous B cells pulsed with BCG, MTB, CA, influenza virus, CMV and EBV peptide pools was measured by determining ^3H-thymidine incorporation (counts per minute [CPM]). (**E**) The frequencies (mean number per million of $CD4^+$ memory T cells) of influenza virus-, CMV-, and EBV-specific memory $CD4^+$ T cells were estimated assuming a Poisson distribution. (**F**) Proliferation, measured by determining ^3H-thymidine incorporation, of memory $CD4^+$ $CCR6^-$ or $CD4^+$ $CCR6^+$ T-cell lines stimulated with autologous B cells pulsed with CMV.

Figure S6

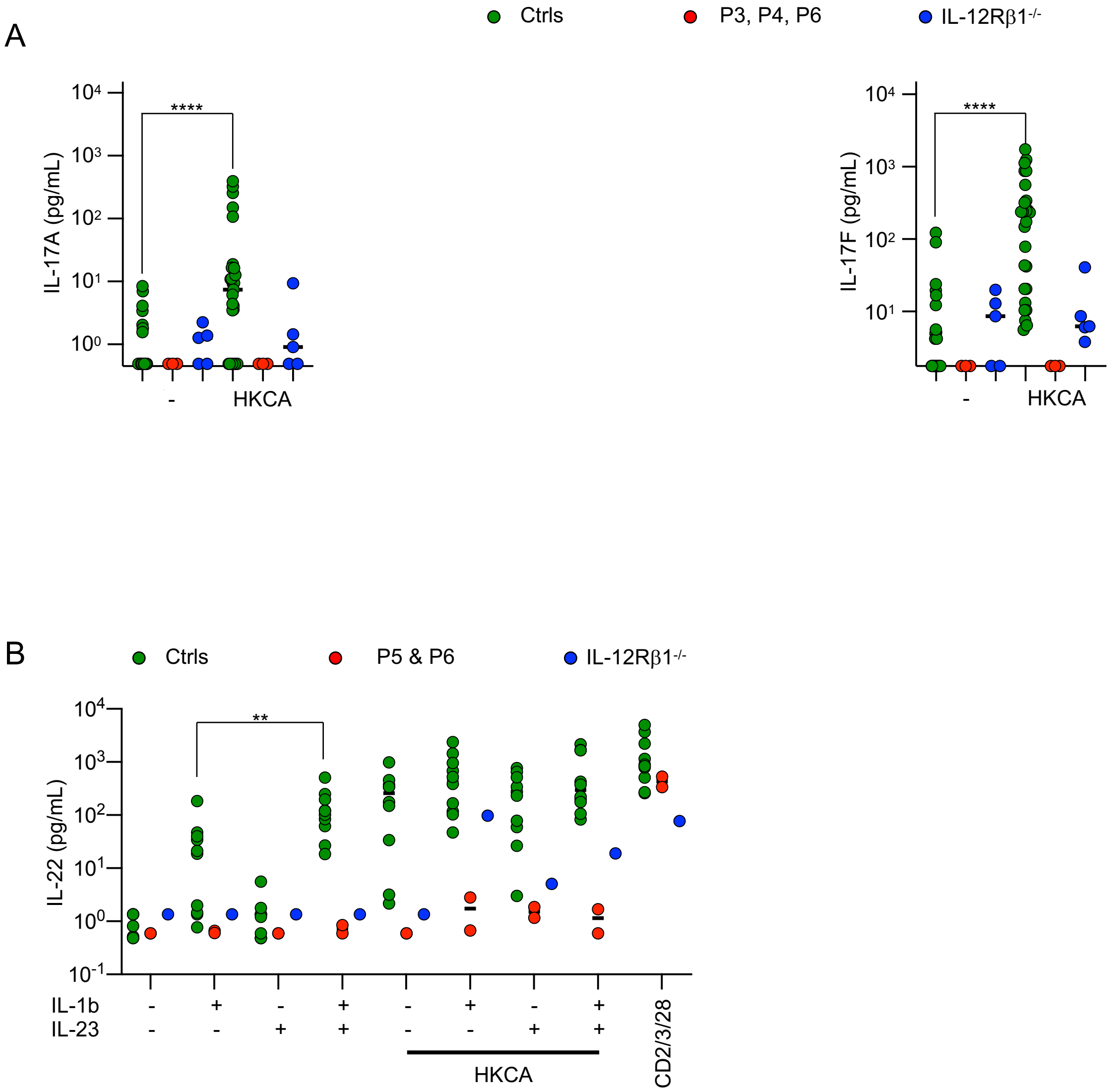
Ctrls
P3, P4, P6
IL-12Rβ1-/-
A
IL-17A (pg/mL)
10⁴
10³
10²
10¹
10⁰
****
-
HKCA
IL-17F (pg/mL)
****
B
Ctrls
P5 & P6
IL-12Rβ1-/-
IL-22 (pg/mL)
10-1
**
IL-1b
IL-23
HKCA
CD2/3/28

Supplementary Figure 6: Impaired *ex vivo* IL-23-mediated production of IL-17 cytokines in patients with inherited IL-23R deficiency

(A) IL-17A and IL-17F secretion by PBMCs, assessed in Legendplex assays on the supernatant, for the indicated individuals (healthy controls, Ctrls; patients P3, P4, and P6; and five IL-12Rβ1-deficient patients). The cells were cultured for seven days, either without stimulation (-) or in the presence of heat-killed *C. albicans* (HKCA). (B) IL-22 secretion by PBMCs, assessed in Legendplex assays on the supernatants, for the indicated individuals (healthy controls, Ctrls; patients P5 and P6; and one IL-12Rβ1-deficient patient). The cells were cultured for five days, either left unstimulated (-) or stimulated with IL-1β (+), and/or IL-23 (+), in the absence or presence of heat-killed *C. albicans* (HKCA), or antibody complexes binding CD2, CD3 and CD28. Nonparametric Mann-Whitney tests were used for the analysis in panels **A-B** (**p<0.01, ****p<0.0001).

Kindred	Patient	Variant	Sex	Year of birth	Follow-up	Mycobacterial disease	Candidiasis		
							**Intestinal tract**	**Oral mucosa**	**Other sites (aphthous stomatitis, genital mucosa, nails, perionyxis, scalp, skin)**
A	P1	p.C115Y	F	1994	Alive	BCG-itis, spontaneous resolution	No	No	No
	P2	p.C115Y	M	2006	Dead	BCG-itis, progression on antibiotic treatment	No	No	No
B	P3	c.367+1G>A	M	2010	Alive	BCG-osis	No	No	No
C	P4	c.1149-1G>A	M	2008	Alive	BCG-itis, spontaneous resolution	Recurrent episodes with complete response to fluconazole, no recurrence on fluconazole prophylaxis	Recurrent episodes with complete response to fluconazole, no recurrence on fluconazole prophylaxis	No
D	P5	p.E269*	M	2015	Alive	BCG-itis, spontaneous resolution		No	No
D	P6	p.E269*	M	2019	Alive	BCG-osis, resolved after antimycobacterial therapy with isoniazid, rifampicin, ethambutol and trimethoprim-sulfamethoxazole		One episode, resolved after fluconazole treatment, no prophylaxis	No

Table S1: Summary of the medical history of patients with inherited IL-23R deficienc

P1	P2	P3	P4	P5	P6
• ADAM32 p.P561T<br>• ADAMDEC1 p.W293R<br>• C11orf80 p.A34dup<br>• HDAC6 p.L1049V<br>• HRCT1 p.P106H<br>• IL23R p.C115Y<br>• INADL g.62231947 G>A<br>• MAGEE2 p.G220R<br>• MPHOSPH9 p.V479I<br>• POLD1 p.R652Q<br>• REXO1 p.G43S	• ADAM32 p.P561T<br>• DNAH3 c.1209-6_1209-5insAGACAG<br>• FN p.R59G<br>• IL23R p.C115Y<br>• MPHOSPH9 p.V479I<br>• PBOV1 p.K91_G92 delinsS<br>• POLD1 p.R652Q<br>• PRKCSH p.E320_E323del<br>• SLC38A10 p.E551Q<br>• TSPAN10 p.T60I	• ATN1 p.Q502del<br>• C11orf80 p.A34dup<br>• C9orf139 p.W41C<br>• DNASE1L2 p.A252G<br>• GJC2 c.-19_-18insGGGGGGGGGGGGGG<br>• IL23R c.367+1G>A<br>• KCNH6 p.N566K<br>• KCNN3 p.Q78_Q80dup<br>• LHX9 c.733+3G>A<br>• RBMS1 c.252-4_252-3del<br>• RUSC1 c.1456+5G>A<br>• SEMG2 p.N272fs<br>• SLC2A10 p.D226N<br>• STIP1 p.A511G<br>• TSC2 p.E1805K	• AR p.Q80dup<br>• ATN1 p.Q497_Q502del<br>• ATP6V0E2 p.R169*<br>• B3GNT3 p.T129M<br>• CA5B p.T244N<br>• CHERP p.G195S<br>• CRIPAK p.T184fs<br>• CRIPAK p.M215fs<br>• CRIPAK<br>• p.C27fs<br>• DDX53 p.L367I<br>• DEPDC1 p.V390L<br>• DLST p.V49I<br>• DTD1 p.A96T<br>• FAM13C p.S38G<br>• GTPBP10 p.L359*<br>• IL23R c.1149-1G>A<br>• MEF2B p.G328R<br>• MUC20 p.A344V<br>• NFE2L1 p.S500_S502del<br>• PCDH15 p.I1755_ p.1760del<br>• PIK3C2G p.Y676H<br>• PPL p.R565W<br>• PRSS27 p.S84P<br>• SPSB2 p.V235A<br>• TRBV20OR9-2 p.R73L<br>• YIPF2 p.I98M<br>• ZNF101 p.R224H<br>• ZNF254 p.T457A	• ACACA c.4416+8C>G<br>• AP1AR c.160-5T>C<br>• ATAD2 p.D275_D277del<br>• CHRDL1 p.P161R<br>• FADS6 p.P15_A16ins18<br>• FAM231B p.W72fs<br>• GPR173 p.R134H<br>• IL23R p.E269*<br>• JUP p.N112S<br>• KIAA1462 p.W1346R<br>• KRTAP16-1 p.S287G<br>• MAL2 p.V40L<br>• NPIPA8 p.A167G<br>• NTN4 p.D468V<br>• PAPSS1 p.Ile519V<br>• PARPBP p.G349V<br>• PIP4K2B c.694-5C>T<br>• TAF2 p.L257R<br>• VIT p.R323Gln<br>• WAC p.R19W	• ALPK1 p.E910D<br>• ATXN3 p.G306fs<br>• CHRDL1 p.P161R<br>• DNAH1 p.R3891W<br>• FADS6 p.P15_A16ins18<br>• FAM231B p.W72fs<br>• FGFR1OP p.K140E<br>• HDAC6 p.E461K<br>• IL23R p.E269*<br>• KIAA1462 p.W1346R<br>• PAPSS1 p.I519V<br>• PPP1R3F p.G77fs<br>• RSF1 p.G699R<br>• TAF1C p.E710K<br>• TBC1D16 p.A217P<br>• THAP4 p.V498I<br>• WAC p.R19W

Table S2: List of all homozygous coding, essential-splicing site and splice-site variants with a CADD above the MSC not present in the homozygous state in GnomAD but detected in the analysis of the exome data of P1 to P6

Experiment	Gene	Genotype
Deep immunophenotyping by CyTOF	*IL12RB1*	p.K305*/p.K305*
Deep immunophenotyping by spectral flow cytometry	*STAT1*	WT/p.R274Q
	STAT1	WT/p.R321G
	STAT1	WT/p.T385M
	STAT1	WT/p.M392T
	IL12RB1	c.783+1 G>A/c.783+1 G>A
	IL12RB1	c.783+1G>A/c.783+1G>A
	IL12RB1	p.K305*/ p.K305*
Single-cell RNA sequencing of primary leukocytes at steady state	*IL12RB1*	c.783+1G>A/ c.783+1G>A
	IL12RB1	p.E542*/ p.E542*
	IL12RB2	p.Q138*/ p.Q138*
	IL12RB2	p.Q138*/ p.Q138*
	STAT1	WT/p.R274Q
Single-cell RNA sequencing on primary leukocytes after IL-23 stimulation	*IL12RB1*	p.Q542*/p.Q542*
	STAT1	WT/p.R274W
PBMC stimulation with BCG	*IL12RB1*	p.E542*/p.E542*
	STAT1	WT/p.R274W
Stimulation of fresh PBMCs with HKCA	*IL12RB1*	p.C196Y/c.1483+182_1619-1073del
Stimulation of freshly thawed PBMCs with HKCA	*IL12RB1*	p.K305*/ p.K305*
	IL12RB1	c.747_752delinsAAAT/Δ12

Table S3: Genotypes of IL-12Rβ1- and IL-12Rβ2-deficient patients and the STAT1-GOF patients included as a control

Gating							Cell ID
Live cells	Single cells	CD66b^{-} CD45^{+}					PBMCs
			CD3^{+} CD19^{-}	TCR γδ^{+}			γδ T cells
				TCR γδ^{-}	CD161 bright		MAIT cells
					CD4^{+} CD8^{-}		CD4^{+} T cells
						CD45RA^{+} CD27^{+}	Naïve CD4^{+} T cells
						Less CD45RA^{+} CD27^{+}	Memory CD4^{+} T cells
					CD4^{-} CD8^{+}		CD8^{+} T cells
			CD3^{-} CD19^{-}	CD14^{-} CD56^{+}			NK
				CD14^{+} CD56^{-}			Monocytes
				CD14^{-} CD56^{-}	HLA DR^{+} CD11c^{+}		mDC
					Less HLA DR^{+} CD11c^{+}	HLA DR bright CD123^{+}	pDC
			CD3^{-} CD19^{+}				B

Table S4: Gating strategy for the assessment of STAT phosphorylation by mass cytometry (CyTOF)

Gating															Cell ID
Live cells	Single cells	CD66b$^-$	Less CD123$^+$ HLADR$^-$	CD45$^+$											PBMCs
					CD3$^-$ CD19$^-$	CD56$^+$									NK cells
							CD56^{bright}								CD56^{bright}
							CD56^{dim}								CD56^{dim}
						CD56$^-$	CD20$^-$ CD14$^-$	HLADR$^+$CD16$^-$		CD123$^+$ CD11c$^-$					pDC
										CD123$^-$ CD11c$^+$		HLADR^{bright} CD123$^-$			mDC
							HLADR$^+$ CD11c$^+$	Less CD38$^+$ CD14$^-$							Monocytes
										CD14$^+$ CD16$^-$					Classical
										CD14$^+$ CD16$^+$					Intermediate
										CD14^{low} CD16$^+$					Non-classical
					CD14$^-$	CD3$^+$ CD19$^-$									Lymphocytes
							TCRγδ$^+$								γδ T
							TCRγδ$^-$	CD4$^+$ CD8$^-$							CD4$^+$ T cells
									CD127$^-$ IL2Rα2$^+$						Treg
									Less CD127$^-$ IL2Rα2$^+$	Less CCR7$^+$ CD45RA$^+$					T_H subpopulation
											CXCR5$^-$	CXCR3$^+$	CCR6$^-$		T_H1
													CCR6$^+$		T_H1*
												CXCR3$^-$	CCR4$^+$	CCR6$^-$	T_H2
														CCR6$^+$	T_H17
											CXCR5$^+$				Tfh
										CCR7$^+$ CD45RA$^+$					CD4$^+$ T Naive
										CCR7$^+$ CD45RA$^-$					CD4$^+$ T CM
										CCR7$^-$ CD45RA$^-$					CD4$^+$ T EM

										$CCR7^{-}$ $CD45RA^{+}$	$CD4^{+}$ T TEMRA
								$CD4^{-}$ $CD8^{+}$			$CD8^{+}$ T cells
										$CCR7^{+}$ $CD45RA^{+}$	$CD8^{+}$ T Naive
										$CCR7^{+}$ $CD45RA^{-}$	$CD8^{+}$ T CM
										$CCR7^{-}$ $CD45RA^{-}$	$CD8^{+}$ T EM
										$CCR7^{-}$ $CD45RA^{+}$	$CD8^{+}$ T TEMRA
								$CD4^{-}$ $CD8^{-}$			DNT cells

Table S5: Gating strategy for deep immunophenotyping by mass cytometry (CyTOF)

Gating											Cell_ID
Live cells	FSC singlets	SSC singlets	D45^{+} CD66b^{-}	CD3^{+} CD19^{-}							PBMCs
					CD3^{+}γδ TCR^{+}	Vδ1^{+}Vδ2^{+}					
						Vδ1^{+}Vδ2^{-}					Vδ1^{+} γδ T cells
						Vδ1^{-}Vδ2^{+}					Vδ2^{+} γδ T cells
						Vδ1^{-}Vδ2^{-}					
					CD3^{+} γδ TCR^{-}	Vα7.2^{+} MR1^{+}					MAIT cells
						Non MAIT	Vβ11^{+} iNKT^{+}				iNKT cells
							Non iNKT	CD4^{+} CD8^{+}			DP T cells
								CD4^{+} CD8^{-}			CD4^{+} T cells
									D25^{+} CD127^{-}		CD4^{+} T Treg
									CD45RA^{+} CCR7^{+}		CD4^{+} T Naïve
									CD45RA^{+} CCR7^{-}		CD4^{+} TEMRA
									CD45RA^{-} CCR7^{+}		CD4^{+} T CM
									CD45RA^{-} CCR7^{-}		CD4^{+} T EM
									CXCR3^{+} CCR6^{+}	CCR4^{-} CCR7$^{+/-}$	T_H1star
									CXCR3^{+} CCR6^{-}	CR4^{-} CCR7$^{+/-}$	T_H1
									CXCR3^{-} CCR6^{+}	CCR4^{+} CCR7$^{+/-}$	T_H17
									CXCR3^{-} CCR6^{-}	CCR4^{+} CCR7$^{+/-}$	T_H2
								CD4^{-}CD8^{+}			CD8^{+} T cells
									CD45RA^{+} CCR7^{+}		CD8^{+} T Naive
									CD45RA^{+}CCR7^{-}		CD8^{+} TEMRA
									CD45RA^{-}CCR7^{+}		CD8^{+} T CM
									D45RA^{-}CCR7^{-}		CD8^{+} T EM
								CD4^{-} CD8^{-}			DN T cells

				CD3^{-} CD19^{+}	CD20^{+} CD38^{-}					B cells
						CD24^{+} CD27^{+}				Memory B cells
						CD24^{-} CD27^{+}				Memory B cells
						CD27^{-}				Naïve B cells
					CD20^{-} CD38^{+}					Plasma cells
				CD3^{-} CD19^{-}	CD14^{+} CD56^{-}	CD14^{+} CD16^{+}				CD16^{+} Monocytes
						CD14^{+} CD16^{-}				CD14^{+} Monocytes
					CD14^{-} CD56^{+}					NK
						CD16dim CD56bright				CD56bright NK
					CD14^{-} CD56^{-}	CD20^{-} $\gamma\delta$ TCR^{-}	CD4– CD8–	CD45$^{+/-}$ CD127^{+} ILC	CRTH2^{+} CD161^{+}	ILC2
									CRTH2^{-} CD117^{+}	ILCP
						CD123$^{+/-}$ HLADR^{+}	CD123^{+} CD11c^{-}			pDC
							CD123^{-} CD11c^{+}			mDC
								CD141^{+} CD1c^{-}		cDC1
								CD141^{-} CD1c^{+}		cDC2

Table S6: Gating strategy for deep immunophenotyping by spectral flow cytometry

<table>
<tr><th colspan="10">Gating</th><th>Cell ID</th></tr>
<tr><td rowspan="9">Live cells</td><td rowspan="9">FSC singlets</td><td rowspan="9">SSC singlets</td><td rowspan="9">Lymphocytes</td><td rowspan="9">D45^{+} CD14^{-}</td><td rowspan="3">CD3^{-} CD19^{-}</td><td rowspan="3">CD56^{+}</td><td colspan="3"></td><td>NK</td></tr>
<tr><td colspan="3">CD56 dim</td><td>CD56 dim NK</td></tr>
<tr><td colspan="3">CD56 bright</td><td>CD56 bright NK</td></tr>
<tr><td rowspan="6">CD3^{+} CD19^{-}</td><td rowspan="2">CD3^{+} $\gamma\delta$ TCR^{+}</td><td colspan="3">Vδ1^{+} Vδ2^{-}</td><td>Vδ1^{+} $\gamma\delta$ T</td></tr>
<tr><td colspan="3">Vδ1^{-} Vδ2^{+}</td><td>Vδ2^{+} $\gamma\delta$ T</td></tr>
<tr><td rowspan="4">CD3^{+} $\gamma\delta$ TCR^{-}</td><td colspan="3">Vα7.2^{+} MR1^{+}</td><td>MAIT</td></tr>
<tr><td rowspan="3">Non MAIT</td><td colspan="2">Vβ11^{+} iNKT^{+}</td><td>iNKT</td></tr>
<tr><td rowspan="2">Non iNKT</td><td>CD4^{+} CD8^{-}</td><td>CD4 T</td></tr>
<tr><td>CD4^{-} CD8^{+}</td><td>CD8 T</td></tr>
</table>

Table S7: Gating strategy for the *ex vivo* evaluation of IFN-γ^{+} and IL-17A^{+} cells after PBMC stimulation with IL-23 or IL-12, in the presence or absence of BCG infection

For each cell subset, the percentages of IFN-γ^{+} and IL-17A^{+} cells were determined.

Gating						Cell ID
Lymphocytes	FSC singlets	Live cells	$CD3^+$	$CD4^+$	$CCR7^+$ $CD45RA^+$	Naive CD4 T^+ cells
					$CCR7^{+/-}$ $CD45RA^-$	Memory $CD4^+$ T cells

Table S8: Gating strategy for the *ex vivo* evaluation of BCG-reactive memory $CD4^+$ T cells

For each cell subset, the percentage of CD40-L^+ and $CD69^+$ cells (reactive cells) was determined, and among the reactive cells, the percentage of IFN-γ^+ cells was determined.

Primer name	Primer sequence 5’ -> 3’
IL23R Exon 3 Forward	GCAATAGCATATTCTTCTGAATC
IL23R Exon 3 Reverse	CACATATGCAGTGTTCGTTCAG
IL23R Exon 7 Forward	GAACACTTTGTTTTCCTAGA
IL23R Exon 7 Reverse	CTGTGCTCAGCAGAAAAGAT
IL23R Exon 10 Forward	CTTATCTTGAATCTAGTGTGAG
IL23R Exon 10 Reverse	CCCAACTCTATTGTCTCATCTC
IL23R Exon 3 Forward XhoI	GACCGCTCGAGGCAATAGCATATTCTTCTGAATC
IL23R Exon 3 Reverse BamHI	GCGGGATCCACATATGCAGTGTTCGTTCAG
IL23R Exon 10 Forward XhoI	GACCGCTCGAGCTTATCTTGAATCTAGTGTGAG
IL23R Exon 10 Reverse BamHI	GCGGGATCCCAACTCTATTGTCTCATCTC
cIL23R delta Exon 3 Forward	ATCCGCCAGATATTCCTGATGAAG
cIL23R delta Exon 3 Reverse	CCATCTTAAAAATTGTGGCTGGTTCTAC
cIL23R c.805G>T Forward	GGAATGTTAAATAATTTGACACC
cIL23R c.805G>T Reverse	GGTGTCAAATTATTTAACATTCC
cIL23R delta exon9 c.1149del Forward	ATTAAAAGAAGGATCTTATTGTTAATACCAA
cIL23R delta exon 9 Reverse	CAGAAGTAAGGTGCCCTGTA
cIL23R Delta Ex 10 Forward	GAAAATAGTGAACTTATGAATAATAATTCCAG
cIL23R Delta Ex 10 Reverse	CCAGTTCGGAATGATCTGTTAAATATC
cIL23R c.1149del Forward	GATCATTCCGAACTGGATTAAAAGAAGGATC
cIL23R c.1149del Reverse	GATCCTTCTTTTAATCCAGTTCGGAATGATC

Table S9 : Sequence of primers used for this study
